# Modeling Cohen Syndrome in Phoenix Cells: VPS13B Loss Causes Organelle Stress, G1/S Delay, and Fibrillary Inclusion Bodies Formation

**DOI:** 10.3390/cells15171535

**Published:** 2026-08-26

**Authors:** Ksenia N. Morozova, Ekaterina R. Wolf, Elena V. Kiseleva, Alexander V. Smirnov, Elena G. Pershina, Inna E. Pristyazhnyuk

**Affiliations:** 1 Institute of Cytology and Genetics, Siberian Branch of the Russian Academy of Sciences, Novosibirsk 630090, Russia; morozko@bionet.nsc.ru (K.N.M.);; 2 Department of Genetics and Genetic Technologies, Sirius University of Science and Technology, Sirius 354340, Russia

**Keywords:** Cohen syndrome, VPS13B, CRISPR-Cas9, COH1, Golgi fragmentation, ultrastructure, ER stress, proteostasis, cell-cycle arrest, fibrillary inclusions

## Abstract

Cohen syndrome, caused by pathogenic variants in *VPS13B*, is characterized by microcephaly, developmental delay, and progressive retinal degeneration, yet the cellular mechanisms linking VPS13B dysfunction to disease pathology remain incompletely understood. Here, we used CRISPR-Cas9 to delete *VPS13B* exons 2–4 in Phoenix HEK293 cells, generating five independent knockout clones. In all mutant lines, VPS13B disruption caused a marked slowing of cell proliferation due to prolongation of the G1 phase. Immunocytochemistry and transmission electron microscopy revealed that *VPS13B* mutations causes Golgi apparatus fragmentation, loss of VPS13B Golgi localization, ER lumen dilation with rigid membrane morphology, mitochondrial damage, impaired autophagic maturation, and the appearance of cytoplasmic fibrillary inclusions located close to ER and absent from control cells. RNA-seq analysis identified 27 differentially expressed genes common to all four mutant clones, including downregulation of genes involved in transcriptional regulation, lipid metabolism, and neuronal signaling, alongside upregulation of the stress-response genes CLU and CDKN1A (p21). While our results do not support classical unfolded protein response activation, they are consistent with a model in which lipid bilayer stress and disrupted ER–Golgi trafficking may play a role in the pathophysiology of Cohen syndrome. Together, these findings demonstrate that VPS13B deficiency results in coordinated defects in organelle homeostasis, proteostasis, and cell-cycle progression, providing new dates for understanding Cohen syndrome pathogenesis.

## 1. Introduction

Cohen syndrome (OMIM 216550) (CS) was first described by Michael Cohen et al. in 1973 [1]. It is an autosomal recessive, multisystem disorder caused by mutations in the *VPS13B* (*COH1*) gene and characterized by intellectual and developmental delay, postnatal microcephaly, hypotonia, metabolic abnormalities, neutropenia, severe myopia, and progressive visual loss due to retinal degeneration [2,3].

**Figure 1:**
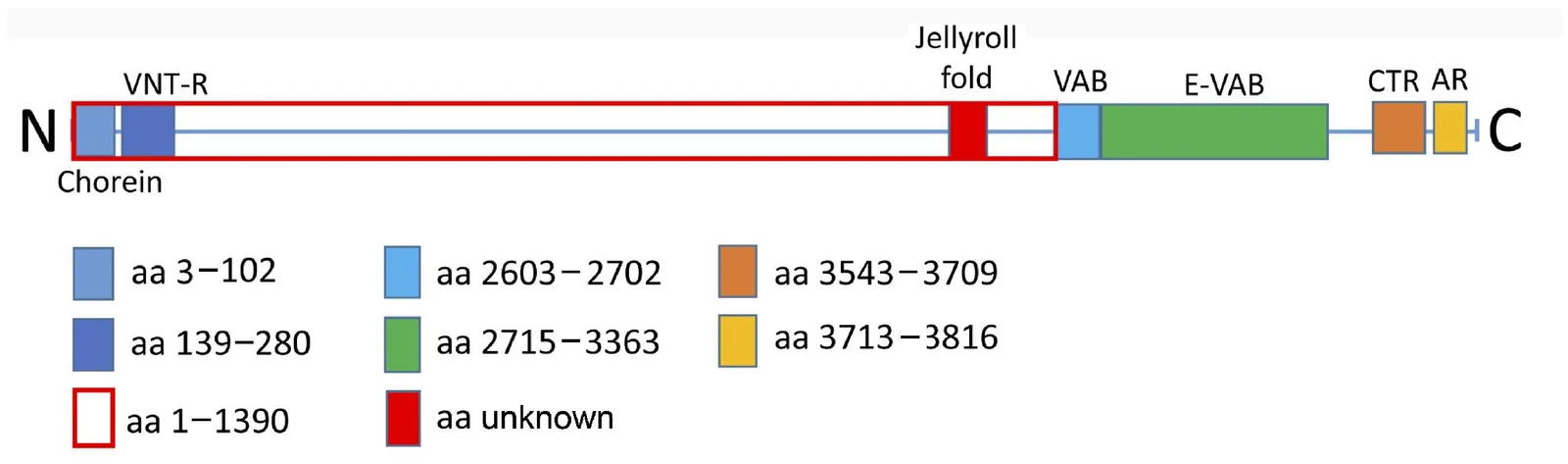
The domain structure of the VPS13B protein. The main domains are marked by the colors: Chorein—Chorein/VPS13 region at the very N-terminus (aa 3–102); VNT-R—a second VPS13-N-terminal region (aa 139–280); Jellyroll domain (the precise domain boundaries are unknown); VAB—a Vps13-adaptor binding domain (aa 2603–2702); E-VAB—an extended VAB domain (aa 2715–3363); CTR—VPS13-C-terminal region a VPS13-C terminal region (aa 3543–3709); and AR—an autophagy-related protein C-terminal domain (aa 3713–3816). The domain organization of the protein is presented according to [10,11].

The VPS13B protein is a member of the vacuolar protein sorting 13 (VPS13) family [4]. In yeast, a single Vps13 protein mediates lipid transport and is essential for vesicular trafficking from the Golgi apparatus (GA), from its trans-Golgi compartment to the late endosome/prevacuolar compartment and for GA retrograde transport [5]. In mammals, this protein family is represented by four members that share structural and functional homology: VPS13A, VPS13B, VPS13C, and VPS13D. All VPS13 proteins are exceptionally large and share a conserved domain architecture [4,6]. Their N-terminal region (approximately residues 1–1390) forms an elongated hydrophobic groove that mediates lipid transport between organelle membranes [7,8], facilitating membrane growth independently of vesicular trafficking [9]. Downstream of the N-terminal channel, a series of domains confers specificity of membrane targeting, including WD40-like repeats [9], the VPS13 adaptor binding domain [4,6], extended VAB domain and, at the terminal region, an autophagy-related protein C terminal domain (known as a pleckstrin homology domain) [10] (Figure 1).

Despite the shared overall architecture, VPS13B is the most evolutionarily divergent member of the family and the closest to yeast Vps13p. Unlike the other mammalian VPS13 proteins, VPS13B contains a Jellyroll fold domain that mediates its association with the GA [11], whereas VPS13A, VPS13C, and VPS13D possess an FFAT motif that targets them to the endoplasmic reticulum (ER) [11].

VPS13B has been shown to localize between cis- and trans-Golgi cisternae [9] and at ER exit site (ERES)–Golgi interfaces [12]. Additionally, VPS13B has been detected at GA to lipid droplet contacts [13] and on recycling endosomes [14]. The GA localization of VPS13B underlies its prominent influence on the structure and function of this organelle. One of the major consequences of *VPS13B* mutations is GA fragmentation [15]. VPS13B deficiency also leads to protein glycosylation defects [16,17], a hallmark of GA dysfunction, as glycosylation is a core post-translational modification carried out within the Golgi [16,17]. Recent studies have revealed a broader involvement of VPS13B in cellular processes beyond Golgi maintenance, including its role in the formation of the secretory pathway from the ER through the GA to the plasma membrane [12] and in ensuring proper mitochondrial division and quality control [17]. Furthermore, cells from patients with *VPS13B* mutations exhibit impaired autophagy, accompanied by the accumulation of autolysosomes with undigested contents [18,19].

Several *VPS13B* knockout cell models have recently been established, including those based on HeLa cells [9,12] and HEK293 cells [13,17]. In the present study, we used the Phoenix cell line, which is derived from HEK293T cells—a human embryonic kidney line [20]. This line was selected because of its ease of cultivation and high transfection efficiency. Importantly, despite their kidney origin, HEK293 cells exhibit several features of neural character, expressing over 60 neuronal genes, including neurofilament proteins and subunits of neuroreceptors and ion channels [21,22,23]. This property is particularly relevant, as the primary manifestations of Cohen syndrome are neurological.

In this study, we generated *VPS13B*-mutant cell lines based on the Phoenix line and characterized their intracellular organization. We demonstrated that VPS13B deficiency leads to profound disruptions of intracellular architecture, accompanied by cellular stress and primarily affecting components of the secretory pathway: fragmentation and dilation of GA cisternae, alterations in ER structure, mitochondrial damage, and accumulation of autophagosomes with undigested contents and fibrillary cytoplasmic inclusions.

## 2. Material and Methods

### 2.1. Plasmids

To disrupt the *VPS13B* gene, a dual-guide CRISPR-Cas9 strategy was employed targeting exons 2 and 4 to delete the intervening genomic region. The exon 2 gRNA (5′–CTCCTTACCTTAAAAGATGC–3′) was cloned into the BbsI sites of pSpCas9(BB) –2A-GFP (PX458) (Addgene #48138) [24] and the exon 4 gRNA (5′–GGTGGTAAGTCAGGATCTGT–3′) was expressed from a separate U6–driven gRNA plasmid (Addgene #41824) [25] (Table S1). Both plasmids were co-transfected into cells, and GFP-positive cells were sorted by flow cytometry 48 h post-transfection.

### 2.2. Cell Culture Conditions and Transfections

The Phoenix cell line, a second-generation retrovirus producer line derived from HEK293 cells, was maintained in growth medium consisting of DMEM/F12 supplemented with 10% fetal bovine serum (FBS), 2 mM L-glutamine (Capricorn Scientific GmbH, Ebsdorfergrund, Germany), and 50 U/mL penicillin/50 µg/mL streptomycin (Thermo Fisher Scientific, Waltham, MA, USA). Cells were kept at 37 °C in a humidified atmosphere containing 5% CO_2_ under standard conditions and were passaged every third day.

For introducing *VPS13B* deletions, transfections with gRNA vectors were performed using Lipofectamine™ 3000 Transfection Reagent (Thermo Fisher Scientific) according to the manufacturer’s protocol. The cells were seeded at a density of 6–9 × 10^4^ cells per cm^2^ in a 24-well plate one day prior to transfection, so that the confluency on the day of transfection was approximately 70%. Then cells were transfected with a 600 µg mixture of plasmids (pSpCas9(BB)/2A-GFP and pRNA_VPS13B) with guide RNA at equimolar concentrations in OPTI-MEM medium for 6 h. On the next day, the cells were harvested and subjected to single-cell sorting (one cell per well) into 96-well plates on a BD FACSAria III (BD Biosciences, Mississauga, ON, Canada) with selection based on green fluorescence. The growth medium in the wells was changed every third day. After colonies reached an appropriate size, they were divided into two aliquots; one was used for screening for the deletion status, while the remaining half was maintained and expanded for downstream experiments. To identify clones carrying the intended deletion of exons 2–4, PCR analysis was performed, and positive clones were selected for further studies.

### 2.3. PCR and Sanger Sequencing

The cell pellet was collected and lysed in PBND buffer with 100 µg/mL Proteinase K (ER-1200, Biolabmix, Novosibirsk, Russia) 30′ at 55 °C. A PCR analysis with specific primers in exon 2 and 4 (Table S1) was used to reveal the clones with target deletion. To confirm the mutation in the second and fourth exons, the Sanger sequencing reactions were performed using the forward and reversed PCR primers in exon 3 and 4 (Table S1).

### 2.4. RNA-Seq

For RNA seq analysis 1–2 × 10^6^ cells were harvested and washed twice with PBS. Total RNA was extracted using SKYEasy RNA Fast Tissue/Cell Kit (ERC451, SkyGen, Guangzhou, China) according to the manufacturing protocol. Sample concentration was evaluated using Qubit RNA HS (Q32852, Invitrogen). Gel electrophoresis was used to verify the integrity of extracted RNA. Four biological replicates were generated for each genotype.

### 2.5. RNA Isolation and RT-PCR

Total RNA was isolated using either the Trizol reagent (Thermo Fisher Scientific, Waltham, MA, USA) or the SKYEasy RNA Fast Tissue/Cell Kit (ERC451, Moscow, Russia, SkyGen), according to the manufacturer’s protocol. First-strand cDNA was synthesized from 1 g of total RNA using the M-MuLV–RH kit (R03-50, Biolabmix, Novosibirsk, Russia) with a random hexamer primer according to the manufacturer’s instructions. Then, cDNA was used as a template for RT-PCR to analyze the gene expression with specific primers for exon 2–4 and exon 25–28 (Table S1).

### 2.6. Transcriptome Analysis

Raw reads were processed using a standardized bioinformatics pipeline in order to avoid batch effects. Initial quality evaluation was conducted with FastQC [26], and then fastP [27] was used for both adapter trimming and the elimination of low-quality bases. The resulting high-quality reads were aligned to the reference human genome (GRCh38.p14) using STAR [28] algorithm. Gene-level expression quantification was managed using featureCounts [29] based on the RefSeq [30] annotation (GCF_000001405.40). Counts were aligned on the exons regions. Differential expression analysis was performed statistically in the R environment using the DESeq2 [31] package. Genes with a |Log2FoldChange| > 1 and a Benjamini–Hochberg adjusted *p*-value below 0.05 were classified as differentially expressed genes (DEGs). RNA-seq data were deposited in the NCBI’s Sequence Read Archive (SRA) (PRJNA1492069).

### 2.7. Cell Doubling Time and Cell Cycle Analysis

For population doubling time measurement, cells were seeded in 24-well culture plates in triplicate for each cell line at a concentration of 10 × 10^4^ cells per well. After 2 days, the cell number was determined using a TC20 automated cell counter (Bio-Rad, Berkeley, CA, USA). The experiment was repeated twice. The doubling time was calculated according to the formula: Td = t/ln(Nt/N0), where Td is the population doubling time, t is the cultivation time, Nt is the cell number at time point t, and N0 is the initial cell number. Population doubling time was compared between Phoenix wild-type cells and Δ2-4VPS13B knockout clones. For each clone, three technical replicates were measured, and the mean of the replicates was used as a single biological data point (n = 9 clones for Phoenix controls; n = 15 clones for Δ2-4VPS13B clones, pooled from three independent experiments). Statistical significance was assessed using an unpaired two-tailed Welch’s *t*-test.

To quantify cell proliferation, the EdU Cell Proliferation Kit (Lumiprobe, Moscow, Russia) was used. The cells were seeded in 12-well culture plates at a concentration of 2 × 10^5^ cells per well. On the following day, EdU was added to the cells for 1.5 h. The cells were then harvested by 0.25% trypsin treatment, and the pellet was fixed with 70% ethanol at 4 °C overnight. The next day, the cells were labeled using a click reaction with fluorescent azides in the presence of a copper catalyst according to the manufacturer’s protocol. For DNA content analysis, the cells were stained with DAPI. Cells labeled in this way were analyzed by flow cytometry using a BD FACSAria with BD FACSDiva™ software (BD Biosciences, Mississauga, ON, Canada) at the Collective Flow Cytometry Center of the ICG SB RAS, Novosibirsk.

### 2.8. Immunocytochemistry and Fluorescence Microscopy

Cells were grown in 24-well plates on 12 mm diameter coverslips and fixed with 4% PFA in PBS for 15 min. Immunofluorescent staining was carried out according to the protocol described earlier [18]. Primary antibodies were applied in a blocking buffer containing 2% BSA and 0.2% Triton X-100 in PBS overnight at 4 °C. Mouse monoclonal antibodies against GM130 (Cat: 610822, BD Biosciences, San Jose, CA, USA) and rabbit polyclonal antibodies against VPS13B (PA5-65371, Invitrogen, Waltham, MA, USA) were used. Signal visualization was performed using secondary antibodies: Goat Anti-Mouse polyclonal IgG (H + L) Fluor488 (S0017, Affinity, Changzhou, China) and Goat Anti-Rabbit IgG (H + L) Secondary Antibody, Alexa Fluor™ 546 (A-11010, Thermo Fisher, Eugene, OR, USA). The cell nuclei were counterstained by DAPI. The stained cells were visualized with a confocal fluorescence microscope LSM 780 NLO with ZEN software, version 20.10 (Zeiss, Jena, Germany).

### 2.9. TEM

Cells grown on Melinex polyester films (175 μm thickness; Agar Scientific, Rotherham, UK) in 12-well plates were pre-fixed directly on the substrate with 2.5% glutaraldehyde in culture medium, followed by fixation in 2.5% glutaraldehyde in 0.1 M sodium cacodylate buffer (pH 7.3) for 1 h at room temperature (RT). Samples were rinsed three times with the same buffer, post-fixed with 1% osmium tetroxide for 1 h, washed twice with double-distilled water, and incubated in 1% aqueous uranyl acetate for 12 h at 4 °C for contrasting. The fixed material was dehydrated through a graded ethanol series (30–100%, 10 min per step), followed by two changes in acetone (10 min each). Dehydrated samples were embedded in EmBed812 resin kit (EMS, Hatfield, PA, USA) using aluminum foil forms and polymerized for 48 h at 60 °C.

Ultrathin sections were cut with a diamond knife (Diatome, Nidau, Switzerland) on an ultramicrotome UC6 (Leica, Vienna, Austria). Sections were examined and imaged using a JEM1400 transmission electron microscope (JEOL, Akishima, Japan) equipped with a Veleta camera and iTEM 5.1 software (both from Olympus, Center Valley, PA, USA). Microscopy was performed at the Center of Collective Use for Microscopic Analysis of Biological Objects, Institute of Cytology and Genetics SB RAS, FWNR-2026-0024.

Morphometric analysis was carried out on ultrathin sections using iTEM 5.1 software (Olympus, Center Valley, PA, USA). For each cell line, a minimum of 20 cells were randomly selected from multiple sections. To avoid observer bias, the analysis was performed under blinded conditions on coded samples.

### 2.10. Colocalization Analysis

Cells were immunostained for VPS13B (rabbit polyclonal antibodies against VPS13B (PA5-65371, Invitrogen, USA) and the cis-Golgi marker GM130 (mouse monoclonal antibodies against GM130 (Cat: 610822, BD Biosciences, San Jose, CA, USA). Confocal images were acquired and colocalization was quantified using the Coloc2 plugin in FIJI/ImageJ, version 1.54p (Fiji GitHub Repository). The following colocalization metrics were merged: Pearson’s correlation coefficient (without threshold), Pearson’s R above Costes auto-threshold, Manders’ overlap coefficients M1 and M2 (above zero intensity of the opposite channel), and thresholded Manders’ coefficients tM1 and tM2 (above Costes auto-threshold of the opposite channel). Pearson’s R calculated only for pixel pairs in which both intensities exceeded the Costes autothreshold. This metric specifically tests whether bright VPS13B signals co-occur with bright GM130 signals. Group differences were assessed using the Kruskal–Wallis H test across all eight groups, followed by the Mann–Whitney U test for the pooled comparison of all control vs. all Δ2-4VPS13B images.

### 2.11. Statistical Analysis

In this study, three to four Phoenix control independent clones and five Δ2-4VPS13B clones (#4, #5, #11, #24, and #25) were considered biological replicates. To quantify GAs area, more than 50 cells per line were analyzed. The area of individual GA objects was drawn manually with a polygon selection tool, and the area was measured using ImageJ software (Bethesda, MD, USA). Overall group differences were evaluated using the Kruskal–Wallis H test. Pairwise comparisons were performed using the Mann–Whitney U test with Bonferroni correction for 28 comparisons.

Cell organelles were measured using iTEM software, version 5.1, in randomly chosen sections. The independent measurements per group for each structure were carried out in a blinded manner. For GA thickness more than 100 independent measurements were made per structure and, for GA length, more than 20 per structure. For mitochondria defects, more than 20 cells were measured in each group and for fibrillary inclusions more than 50 cells were calculated. The percentage of mitochondria with structural defects was quantified from electron micrographs in three control clones and five Δ2-4VPS13B clones. For each section, the number of defective mitochondria and total mitochondria were counted, and the percentage of defective mitochondria was calculated. The control and Δ2-4VPS13B groups comparison was statistically evaluated by the Kruskal–Wallis test. *p* values ≤ 0.05 were considered statistically significant.

The proportion of cells containing fibrillary inclusion bodies structures was compared between control and Δ2-4VPS13B groups. A parametric test for proportions was performed using the Mann–Whitney U test. Each section was treated as an independent replica.

Population doubling time was compared between Phoenix control cells and Δ2-4VPS13B clones. For each clone, three technical replicates were measured, and the mean of the replicates was used as a single biological data point (n = 9 clones for Phoenix control; n = 15 clones for Δ2-4VPS13B, pooled from three independent experiments). Statistical significance was assessed using an unpaired two-tailed Welch’s *t*-test. Differences were considered statistically significant at *p* < 0.05.

Cell cycle distribution (G1, S, and G2 phases) was compared between Phoenix control and Δ2-4VPS13B clones. Each clone was measured once per experiment, and data from five independent experiments were pooled. Statistical significance was assessed by two-way ANOVA with group and experiment as factors, followed by Bonferroni correction for multiple comparisons across the three cell cycle phases. Differences were considered statistically significant at *p* < 0.05.

For the colocalization analysis all metrics were compared across the three control groups and five Δ2-4VPS13B. Group differences were assessed using the Kruskal–Wallis H test across all eight groups, followed by the Mann–Whitney U test for the pooled comparison of all control vs. all Δ2-4VPS13B images.

Statistical analyses were performed using Python (version 3.10) with the SciPy library (version 1.11.4; scipy.stats). Data processing was facilitated using the Biomni Lab platform https://biomni.phylo.bio/, accessed on 1 July 2026 [32].

## 3. Result

### 3.1. Experimental Design

The tight connection between VPS13B and the Golgi apparatus (GA) underlies its remarkable effect on Golgi structure and function. Fragmentation of the Golgi apparatus, a hallmark of VPS13B dysfunction, has been observed across various cell types [15,18,33]. However, this is not the only intracellular alteration arising from VPS13B deficiency. In our earlier work, we characterized the ultrastructure of neurons differentiated from induced pluripotent stem cells (iPSCs) generated from patients with pronounced symptoms of Cohen syndrome harboring compound heterozygous variants in the *VPS13B* gene [18]. Our findings revealed that this condition is associated with prominent ultrastructural defects, such as GA fragmentation, endoplasmic reticulum (ER) stress, disrupted mitochondrial and plasma membrane morphology, and autophagic dysfunction, including an elevated abundance of acidic compartments and the buildup of large autophagic vacuoles with undigested contents. However, iPSC-derived neurons present considerable limitations for mechanistic studies due to their low yield, prolonged differentiation time, and restricted suitability for genetic manipulation and functional assays.

To overcome these constraints, we turned to human embryonic kidney 293 (HEK293) cells as a more experimentally convenient model enabling investigation at the level of whole cell populations. Although not neural in origin, HEK293 cells exhibit several features of neural character [21,23] and are widely used as a surrogate system for studying intracellular trafficking and organelle organization [34]. Their high transfection efficiency, rapid proliferation, and amenability to clonal selection [35] make them particularly well-suited for CRISPR/Cas9-mediated knockout and subsequent functional characterization. Importantly, unlike iPSC-derived neurons, which yield limited and heterogeneous cell numbers, HEK293 cells provide a homogeneous and scalable population, allowing quantitative analyses of organelle morphology, autophagic flux, and GA dynamics across large cell cohorts with sufficient statistical power.

### 3.2. Generation of Mutant Phoenix Cell Line

To generate Phoenix clones with disrupted *VPS13B* gene, we introduced two separate double-strand breaks in the *VPS13B* gene and target sites were selected within exons 2 and 4, which encode the N-terminal portion of the hydrophobic groove of the protein. Guide RNAs were designed to target the beginning of exon 2, in close proximity to the start codon, and the end of exon 4 (Supplementary Table S1). The gRNAs were cloned into separate plasmids, one of which carried Cas9 and GFP as a selectable marker. Following Lipofectamine transfection of HEK293 (Phoenix) cells, approximately 100 clones were isolated by FACS based on GFP fluorescence. PCR screening with primers flanking the targeted deletion of exons 2–4 identified five independent clones carrying homozygous deletions of the target region (Figure S1A,B). Sanger sequencing revealed precise end-joining of the double-strand break ends in two of the five clones (hereafter designated Δ2-4VPS13B#11 and Δ2-4VPS13B#24). In the Δ2-4VPS13B#25 clone, repair of the double-strand break occurred with a single-nucleotide insertion (hereafter designated Δ2-4VPS13B#25). In the Δ2-4VPS13B#4 and Δ2-4VPS13B#5 clones, break repair led to the formation of a compound heterozygous mutant variant (Figure S1C).

RT-PCR analysis of these clones, using primers targeting different exons of the *VPS13B* transcript, revealed the synthesis of a truncated transcript spanning most of the gene, with the exception of the deleted region (Figure S1D). Translation of this transcript likely initiates from an alternative start codon located in exon 5. RNA-seq analysis of these clones showed that the normalized number of reads aligned to the *VPS13B* region was approximately equal to that observed in the control samples, in contrast to Δ2-4VPS13B#25 clone. Δ2-4VPS13B#25 clones had the lowest normalized read count compared to the other clones and controls (Figure S1E). By the way, RT-PCR also reveals that all four mutant clones carry a large genomic deletion spanning exons 2–4 (~82.6–82.9 kb), generated by dual-guide CRISPR-Cas9 targeting exon 2 (gRNA1) and exon 4 (gRNA2). As seen at the Figure S1F, control shows uniform coverage across exons 2–6, whereas mutant clones lack detectable reads at exons 2–4. Coverage resumes at exon 5 in Δ2-4VPS13B#4, Δ2-4VPS13B#5, and Δ2-4VPS13B#24, consistent with residual transcription from the intact downstream portion of the gene. The deletion removes the canonical start codon (ATG, exon 2) and is predicted to result in either nonsense-mediated decay or, if a downstream ORF is utilized, translation initiation from an alternative ATG within exon 5, producing a severely truncated and non-functional protein. Δ2-4VPS13B#25 shows markedly reduced overall *VPS13B* expression compared to other clones, suggesting additional post-transcriptional destabilization of the truncated transcript. It is possible that the single-nucleotide insertion introduced during break repair caused a frameshift, leading to nonsense-mediated decay (NMD). As a result, RNA expression in this clone was reduced approximately 1.5-fold. Thus, we obtained a panel of five clones with impaired VPS13B function (Figure S1A). In four of these clones, the N-terminal portion of the protein was disrupted, which is expected to compromise lipid transport through the hydrophobic groove. In the fifth clone, the mutation likely resulted in a near-complete knockout of *VPS13B* due to NMD.

### 3.3. Characteristics of Mutant Phoenix Cell Line

#### Golgi Apparatus

**Figure 2:**
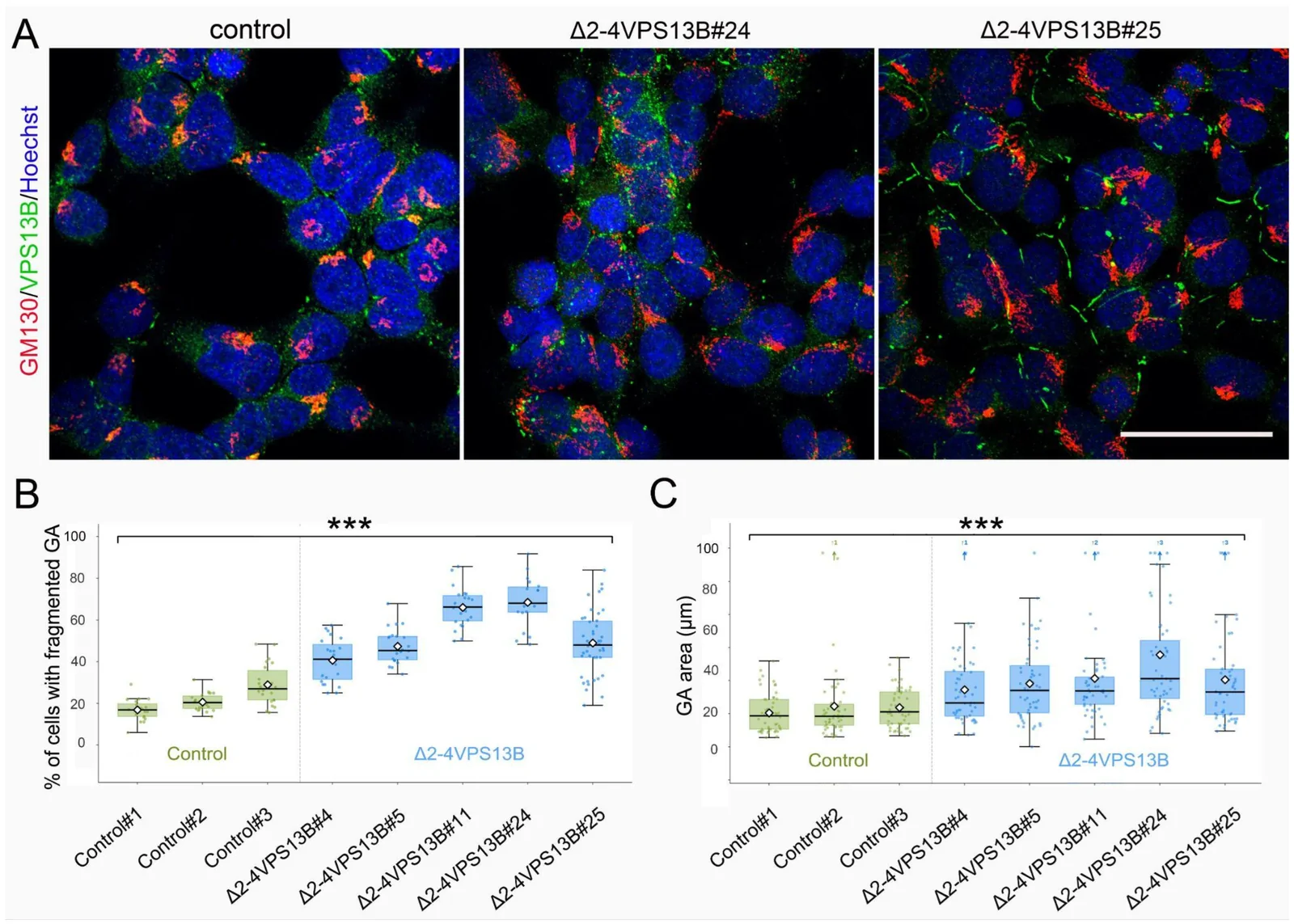
Morphological changes of Golgi apparatus (GA) in Phoenix Δ2-4VPS13B clones by ICH analysis. (**A**) Immunocytochemical analysis of Phoenix Δ2-4VPS13B clones demonstrates a compact GA in control Phoenix cells and dispersed GA in Phoenix Δ2-4VPS13B clones. The nuclei are stained with Hoechst 33258 (blue), GA is stained by GM130 (red), and the VPS13B in green. Scale bar: 50 µm. (**B**) Quantitative analysis of the percentage of cells with dispersed GA in Δ2-4VPS13B clones in comparison with control Phoenix cells per microscope field. The Kruskal–Wallis test revealed a highly significant difference among the eight groups (H = 141.30, *p* = 2.72 × 10^−27^). (**C**) Quantitative analysis of the GA area in Δ2-4VPS13B clones in comparison with control Phoenix cells. The Kruskal–Wallis test revealed a highly significant difference among the eight groups. Bar plots represent the mean ± SEM. Statistically significant differences between groups are marked as *** = *p*-value < 0.001.

**Figure 3:**
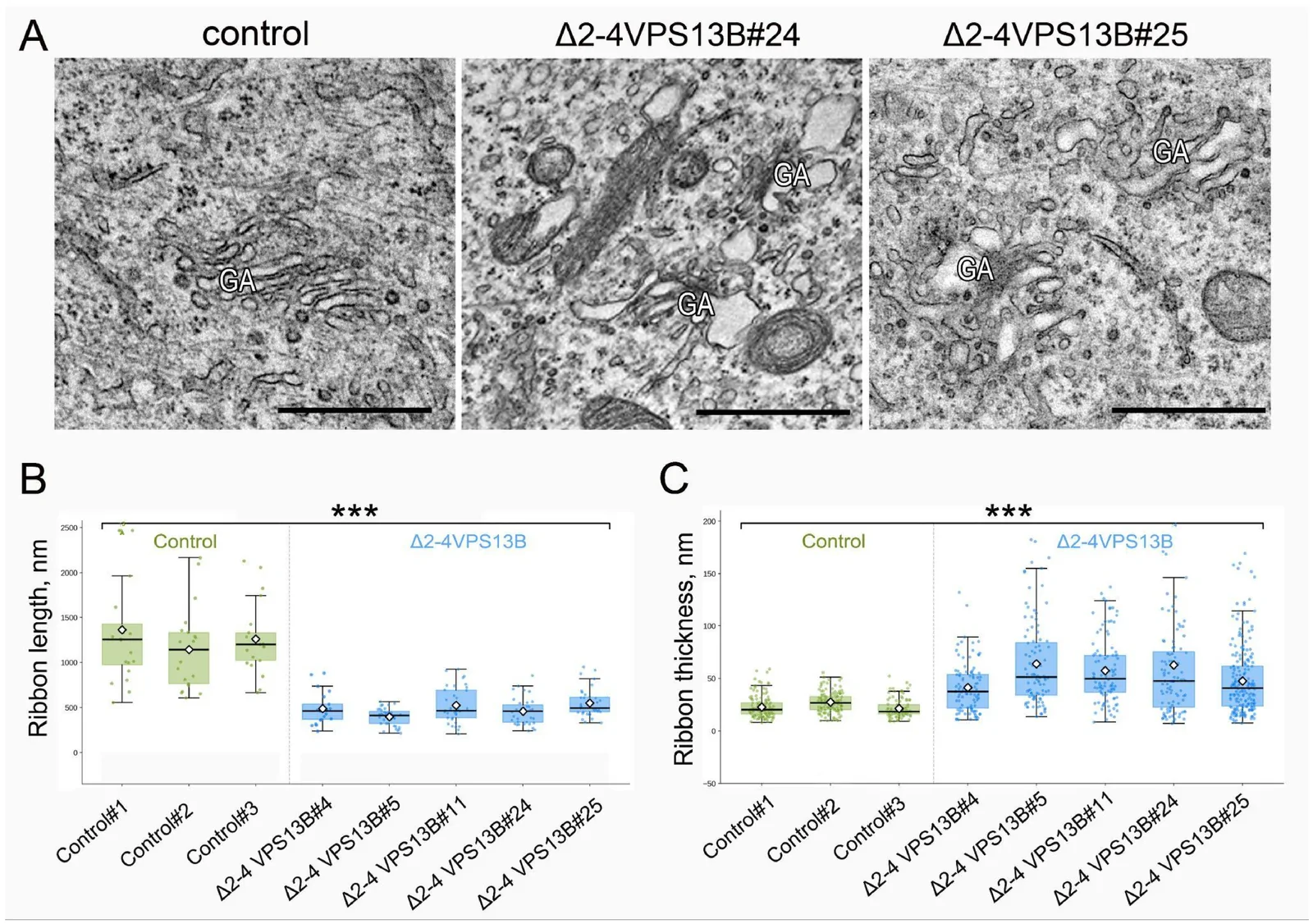
Morphological changes of GA in Phoenix Δ2-4VPS13B clones by TEM analysis. Electron micrographs of the Golgi apparatus in the cytoplasm of control Phoenix cells and mutant Phoenix cells (**A**) demonstrates dictyosome stacks that are ordered and continuous in control cells and short, fragmented, and swollen in mutant Δ2-4VPS13B cells. Scale bar: 1 µm. Quantitative analysis of GA morphological parameters in Δ2-4VPS13B cells and CS patients: ribbon length (**B**) and thickness (**C**). The Kruskal–Wallis test revealed a highly significant difference among the eight groups (H = 128.97, *p* = 1.03 × 10^−24^ for GA length and H = 275.98, *p* < 10^−55^ for GA thickness). Bar plots represent the mean SEM. Statistically significant differences between groups are marked as *** = *p*-value < 0.001.

These disturbances in the *VPS13B* gene are accompanied by cellular stress and affect the components of the secretory pathway, resulting in GA fragmentation and expansion of the GA cisternae, as detected in mutant cells (Figure 2). GM130 staining of the GA reveals fragmented GA in most Δ2-4VPS13B cells (Figure 2B), and the GA area is also significantly increased in these cells (Figure 2C). Transmission electron microscopy revealed long, continuous GA dictyosomes in control Phoenix cells and short, fragmented, and swollen dictyosome stacks in mutant cells (Figure 3A). The GA ribbon length was significantly decreased (Figure 3B), whereas their thickness was increased (Figure 3C). Thus, fragmented and swollen GA was present in all mutant Δ2-4VPS13B clones, representing the most characteristic ultrastructural feature of cells with mutations in *VPS13B*.

### 3.4. Localization of VPS13B

It has previously been shown that VPS13B is a resident protein of the GA and strongly co-localizes with the cis-Golgi matrix protein GM130 [36]; however, when the structure of this protein is disrupted, it loses its association with this organelle and becomes distributed throughout the cytoplasm. This effect has been demonstrated for several mutations, primarily located in the region adjacent to the VAB domain, as well as for those disrupting the C-terminal PH domain [10,12,37].

Therefore, we decided to determine whether the localization of VPS13B is altered due to the deletion of exons 2–4, corresponding to exons 2–4, which encode the N-terminal portion of the hydrophobic domain of the protein. To this end, we performed simultaneous immunocytochemical staining of cells with antibodies against the GM130 protein and antibodies against VPS13B.

**Figure 4:**
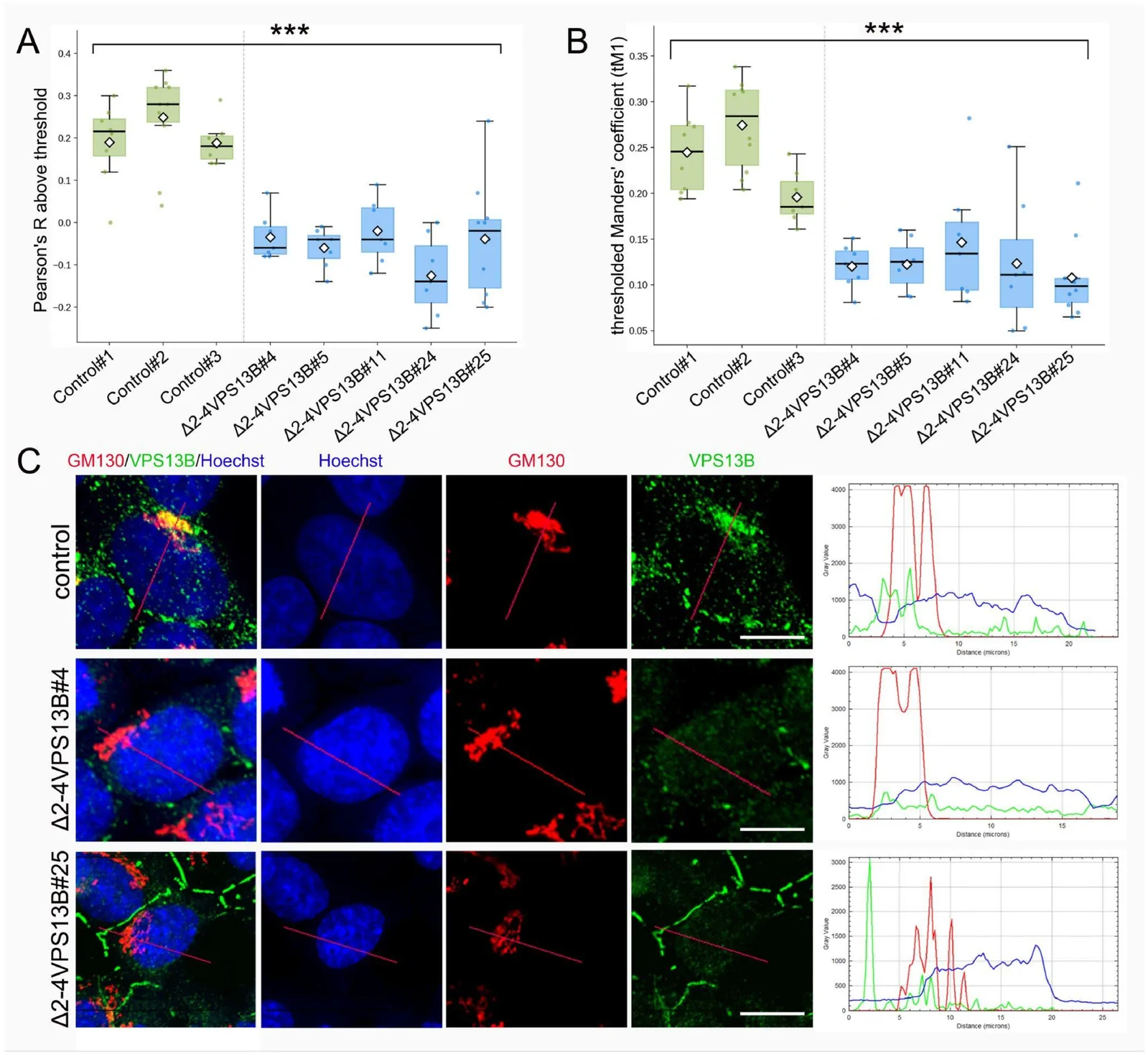
VPS13B and GA localization. (**A**) Pearson’s correlation coefficient of the VPS13B and GM130 bright signals (Pearson’s R above threshold). (**B**) The thresholded Manders’ coefficient tM1, which represents the fraction of bright VPS13B signal overlapping with the GA. Box plots with individual data points and mean (white diamond) are shown. Horizontal brackets indicate pooled Mann–Whitney U test, *p* < 0.001. Bar plots represent the mean ± SEM. Statistically significant differences between groups are marked as *** = *p*-value < 0.001. A dashed vertical line separates control and experimental groups. (**C**) Line-scan shows VPS13B (green), GM130 (red) and Hoechst 33258 (blue) staining localization in Phoenix controls cells, Δ2-4VPS13B#4, #5, #11, and #24 clones and Δ2-4VPS13B#25 clone. Scale bar: 1 µm.

Analysis of colocalization between these stainings revealed that Pearson’s R coefficient, based on pixel-by-pixel comparison, was approximately 0.39 in controls (moderate positive correlation, indicating partial overlap between VPS13B and GM130), whereas in Δ2–4VPS13B clones it was approximately 0.20 (markedly reduced correlation). However, when analyzing the overlap in the localization of bright signals (Pearson’s R above threshold), the difference became obvious. In controls, R > 0 (+0.21), indicating that bright VPS13B signals colocalize with bright GM130 signals at the Golgi. In contrast, in Δ2–4VPS13B clones, R < 0 (−0.05), indicating that bright VPS13B signals localize outside GM130-positive areas, i.e., bright pixels from VPS13B staining were not associated with the Golgi but rather with the cytosol or other compartments (Figure 4A). The thresholded Manders’ coefficient tM1, which represents the fraction of thresholded (bright) VPS13B staining that overlaps with the Golgi apparatus, was approximately 0.24 in controls, indicating that about one quarter of bright VPS13B signal was localized to the Golgi. In Δ2–4VPS13B clones, tM1 was approximately 0.12, nearly twofold lower (Figure 4B). Thus, we have demonstrated that the truncated protein loses its targeting to GA.

Furthermore, in clone Δ2–4VPS13B#25, loss of the transcript results in VPS13B relocation to the cell periphery, where it resides directly beneath the plasma membrane (Figure 4C). This effect has been previously observed in cells with RAB6 knockdown [15], suggesting that the loss of VPS13B localization was due to impaired recruitment of the protein to the Golgi apparatus rather than disruption of the protein structure.

### 3.5. Characteristics of Cell Cycle Parameters of Phoenix Cell Line

**Figure 5:**
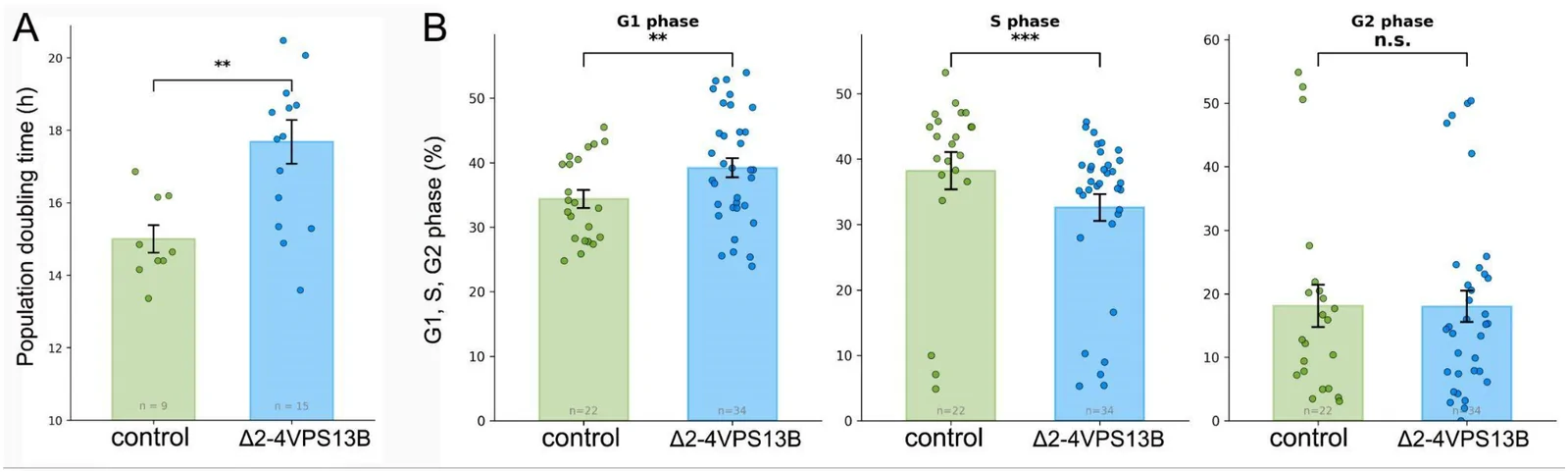
Characteristics of cell population growth. (**A**) Population doubling time in Δ2-4VPS13B knockout clones. Bar plot shows mean population doubling time (h) for Phoenix control (green) and Δ2-4VPS13B knockout (blue) clones. Statistical significance was determined as ** *p* = 0.0011. Data are presented as mean ± SEM, individual data points are shown. (**B**) Cell cycle distribution in Δ2-4VPS13B clones. Bar plots show the percentage of cells in G1, S, and G2 phases for three independent Phoenix controls (green) and five Δ2-4VPS13B (blue) clones. The individual data points are shown. Error bars indicate SEM. Statistical significance was determined as *** *p* < 0.001, ** *p* < 0.01; n.s., not significant.

The generated Phoenix clones with a deletion of the *VPS13B* region divided significantly slower, which is consistent with previously reported reduction in the proportion of actively proliferating neural progenitors in neurospheres derived from iPSCs of Cohen syndrome patients [19]. Assessment of the proliferative activity of the generated clones across five independent experiments revealed a significant (*p* ≤ 0.01) increase in population doubling time compared to control Phoenix lines (15.00 ± 1.15 h vs. 17.68 ± 2.34 h) (Figure 5A).

To determine at which stage the cell cycle is delayed, we examined cell proliferation and DNA replication using click chemistry with EdU (5-ethynyl-2′-deoxyuridine) added to the culture medium [19] (Figure 5B). This analysis revealed that Δ2-4VPS13B clones have an increased proportion of cells in G1 phase (+4.8%, *p* = 0.006) and a decreased proportion in S phase (−5.6%, *p* = 0.0003), consistent with the increased population doubling time. The proportion of cells in the G2 phase remained unchanged. A possible explanation for this phenotype may be related to the disruption of GA structure upon *VPS13B* knockout, which has been observed both in patient-derived neurons and fibroblasts [18,19,36] and in VPS13B-deficient models [9,12,36,38]. Sin and Harrison (2016) demonstrated that the mammalian GA grows by cisternal elongation beginning at mid- to late-G1 phase, concomitantly with cell size increase, and continues to elongate toward G2 phase; this process requires lipids transported from the ER to the GA via transitional ER (tER) sites [39]. Disruption of lipid transport by VPS13B may thus slow the growth of Golgi cisternae, thereby delaying passage through the G1/S checkpoint.

### 3.6. TEM Analysis of Mutant Phoenix Cell Line

In our previous study on iPSC-derived neurons from SC patients, we observed pronounced alterations in intracellular organization, affecting not only the Golgi apparatus but also mitochondrial and ER structure, as well as inducing dilation of the intermembrane space of double-layered membrane envelopes and accumulation of autophagosomes containing undigested membranous content [18].

**Figure 6:**
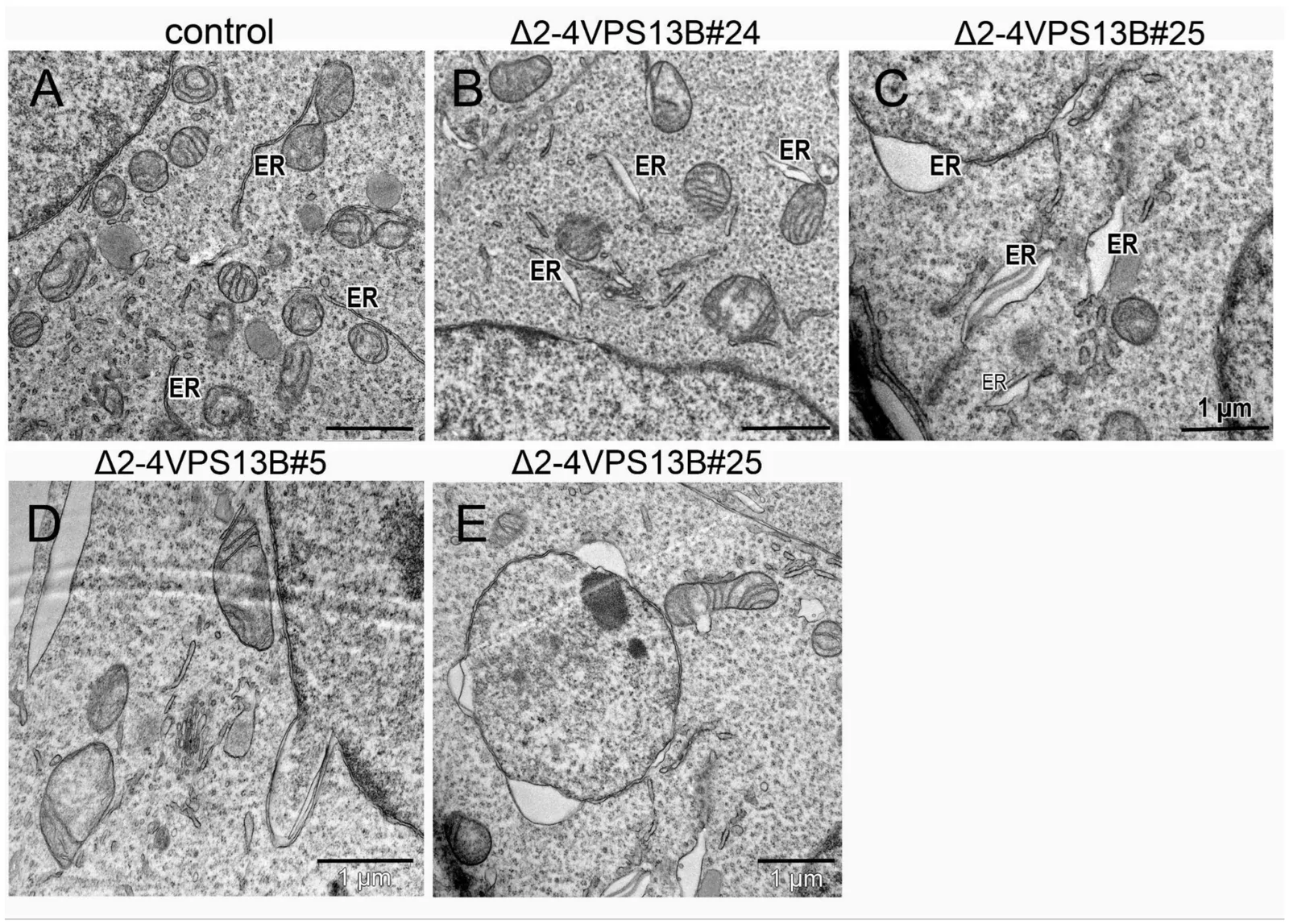
Ultrastructural aspects of ER stress in the mutant Phoenix cell line. There are long narrow ER membranes in control Phoenix line (**A**) and straight rigid ER membranes and dilated ER cistern lumen in mutant Δ2-4VPS13B#4, #5, 11, #24 and #25 (**B**,**C**) clones. These ER membrane changes also extended to the nuclear envelope (**D**,**E**). Scale bar: 1 µm. ER—endoplasmic reticulum.

In the present study, we performed a detailed ultrastructural analysis of Δ2-4VPS13B clones. TEM analysis of mutant Δ2-4VPS13B lines revealed ER lumen dilation and membrane alterations, with the membranes appearing rigid and inflexible (Figure 6). Notably, these ER membrane changes also extended to the nuclear envelope (Figure 6C,D). Several mechanisms could potentially underlie these alterations, including ER stress caused by the accumulation of unfolded proteins in the organelle lumen (unfolded protein response, UPR) [40], lipid bilayer stress (LBS) associated with lipid imbalance in ER membranes [41], or disruption of membrane tension due to impaired lipid transport between the ER and the Golgi apparatus [42,43]. A definitive answer cannot be done at this stage, and further studies are required to explore this phenomenon.

**Figure 7:**
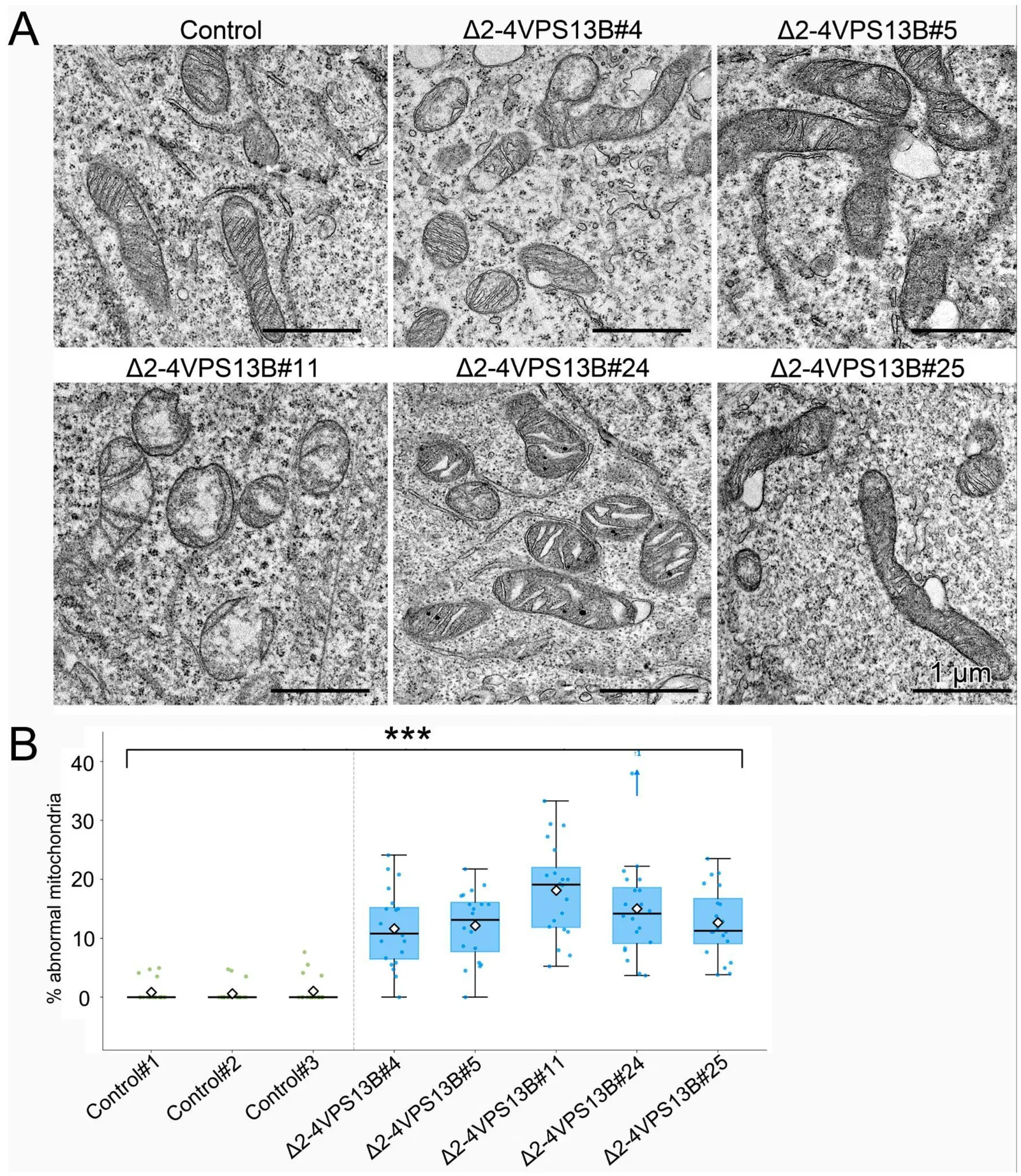
Mitochondria defects in the mutant Phoenix cell line. (**A**) TEM images of mitochondria in control Phoenix cells and mutated Δ2-4VPS13B clones. Control cell mitochondria have normal morphology, but in *VPS13B*-mutated cells mitochondria have numerous anomalies: empty matrix area (without cristae), swollen cristae and mitochondria membrane protrusions. Scale bar: 1 µm. (**B**) Quantitative analysis of morphological parameters in control Phoenix and mutant Δ2-4VPS13B clones. Box plots with individual data points and mean (white diamond) are shown. Horizontal brackets indicate pooled Mann–Whitney U test, *p* < 0.001. Bar plots represent the mean ± SEM. The Kruskal–Wallis test revealed a highly significant difference among the eight groups. ***-*p* = 1.25 × 10^−20^.

Mutation in the *VPS13B* gene causes profound mitochondrial morphological changes. Mitochondria lose integrity, with membrane bulging and disruption. The matrix contains cristae-free empty areas, and swollen cristae were also observed in some cases (Figure 7). At the same time, control mitochondria had a light matrix and narrow, regular cristae. The observed changes in mitochondrial morphology—including shortened or swollen organelles, loss of cristae, and outer membrane disruption—are characteristic of secondary mitochondrial injury. This is in contrast to primary mitochondrial fission failure, where fusion continues while fission is blocked, resulting in elongated or giant mitochondrial networks [44,45]. According to our morphometric analysis, the total proportion of mitochondria exhibiting various morphological defects ranged from approximately 10% to 20% of the entire organelle population in mutant clones. This is consistent with recent findings that lipid transfer by VPS13B is required for membrane fission and that, in the absence of this protein, cells exhibit impaired mitophagy and accumulation of abnormally elongated and fused mitochondria with reduced membrane potential [17]. However, TEM morphology alone is insufficient to demonstrate defective mitophagy. Definitive demonstration of impaired mitophagy requires functional assays such as LC3-II turnover with lysosomal inhibitors, PINK1/Parkin recruitment assays, or mitophagy reporters.

Impairment of autophagy and lysosomal function has been observed both in the context of *VPS13B* mutation [18,33] and following *Rab6* knockout, with which VPS13B forms a functional complex, likely due to disrupted cathepsin trafficking to autophagosomes [15,46]. Accumulation of autolysosomes with undigested membranous content has previously been reported in neurons derived from iPSCs of patients with CS [18,33].

**Figure 8:**
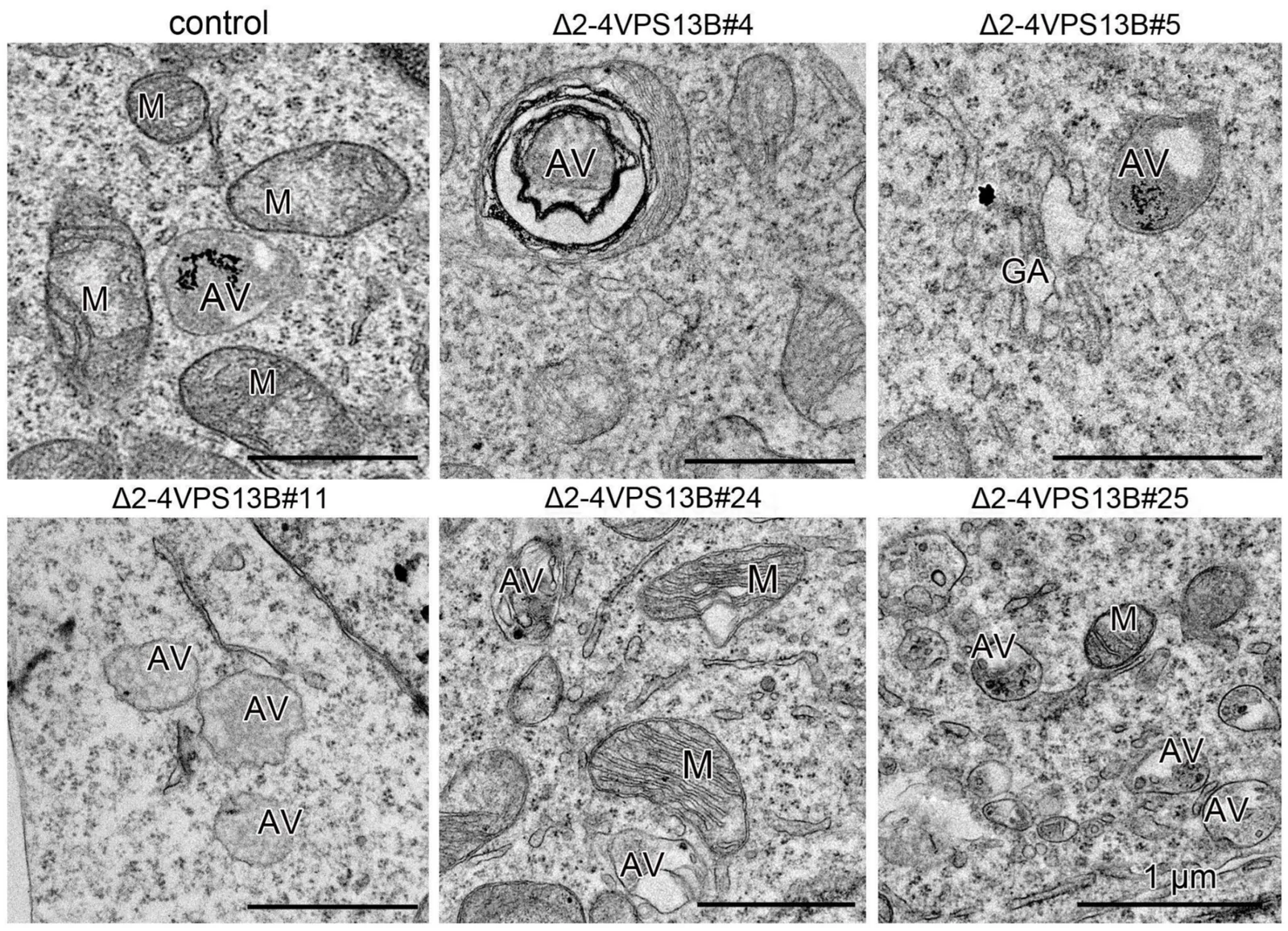
TEM images of autophagic vacuoles in control Phoenix cells and mutated Δ2-4VPS13B clones. Primary and secondary lysosomes (autophagosomes, containing membrane structures of mitochondria and ER fragments at various stages of digestion) in the cytoplasm of control and mutant Δ2-4VPS13B#4, Δ2-4VPS13B#5, Δ2-4VPS13B#11, Δ2-4VPS13B#24 and Δ2-4VPS13B#25 cells. AV—autophagic vacuoles, M—mitochondria, GA—Golgi apparatus. Scale bar: 1 µm.

In Phoenix Δ2-4VPS13B cells, we also observed signs of autophagy impairment. TEM analysis showed accumulation of autolysosomes in the Δ2-4VPS13B cell cytoplasm. In the cytoplasm of mutant cells, primary and secondary lysosomes were detected. Secondary lysosomes were of the autophagic type, containing membrane structures and organelle remnants at various stages of digestion (Figure 8). Mutant Δ2-4VPS13B#5 and Δ2-4VPS13B#11 cells predominantly exhibited primary lysosomes, similar to those observed in control cells. In Δ2-4VPS13B#4 cells, secondary lysosomes were frequent. In Δ2-4VPS13B#24, autolysosomes were detected, with easily recognizable segregated mitochondria and ER fragments inside. At the same time, in Δ2-4VPS13B#25 cells, accumulation of numerous small lysosomes was observed.

**Figure 9:**
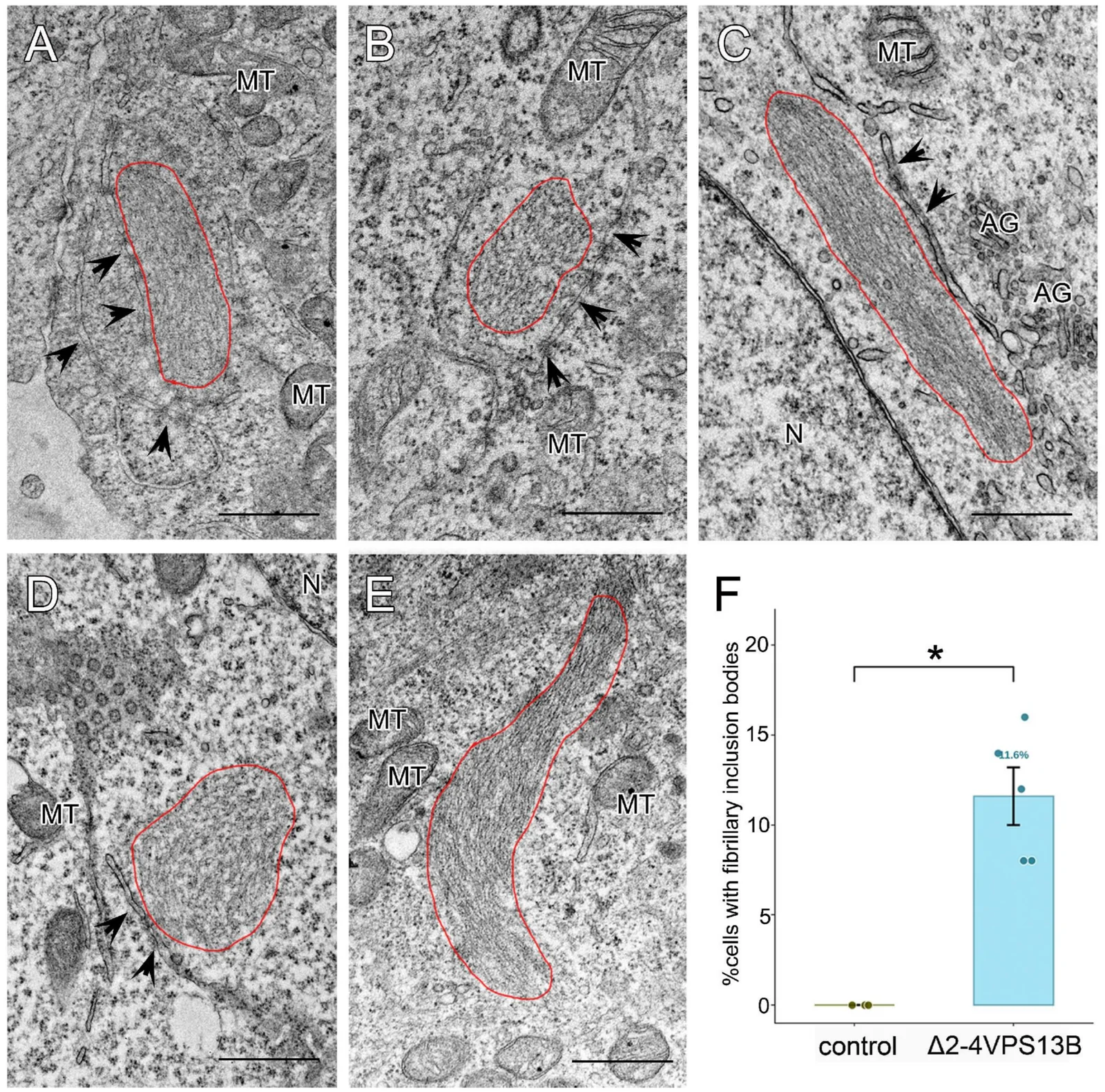
Fibrillary inclusion bodies in Δ2-4VPS13B cell clones. Δ2-4VPS13B#4 (**A**), Δ2-4VPS13B#5 (**B**), Δ2-4VPS13B#11 (**C**), Δ2-4VPS13B#24 (**D**) and Δ2-4VPS13B#25 (**E**) cell lines. ER and annulate lamellae (arrowheads) are often detected near the fibrillary inclusion bodies. N—nucleus, MT—mitochondria, AG—Golgi apparatus, arrowheads mark annulate lamellae, and fibrillary inclusions are surrounded by a red line. Scale bar: 1 µm. (**F**) Quantitative analysis of morphological parameters in control Phoenix and mutant Δ2-4VPS13B clones. Data are presented as mean ± SEM, individual data points are shown. Mann–Whitney U test, * *p* < 0.05.

However, a novel finding we observed in Phoenix Δ2-4VPS13B cells is the appearance of fibrillary inclusion bodies (F-bodies) in the cytoplasm of these cells (Figure 9). Quantitative analysis of morphological parameters in control Phoenix cells and mutant Δ2-4VPS13B clones revealed that fibrillar structures are significantly more frequent in Δ2-4VPS13B cells. While control clones showed no fibrillar structures, Δ2-4VPS13B clones exhibited fibrillary inclusion bodies in 8–16% of cells. In this study, we were unable to determine the nature of these inclusions. We hypothesized that these could be amyloid-like bodies, similar to A-bodies, the stress-inducible physiological amyloid-like structures that sequester proteins into an amyloid-like state in response to heat shock, hypoxia/acidosis, and transcriptional/proteotoxic stress [47]. They share properties with pathological amyloids, being dense, frequently containing fibrillar proteins, and insoluble, yet they form rapidly and reversibly, representing a protective sequestration mechanism [48,49]. However, staining with Thioflavin T, a standard dye widely used to detect amyloid fibrils, did not yield a positive result. F-bodies in Δ2-4VPS13B cells present as inclusions of fibrillar proteins, similar in morphology to protein aggregates formed by N-terminal fragments of huntingtin inside patient neurons [50] and, like these inclusions, they are located in close proximity to the ER. In our case, annulate lamellae (AL) were often detected near the F-bodies. Annulate lamellae are specialized compartments of the ER consisting of ER cisternae with pore complexes that are structurally identical to nuclear pore complexes [51,52]. These unique organelles are found primarily in embryonic cells, germ cells, and rapidly dividing tumor cells. AL are a typical organelle of the HEK293 cell line. In mutant Δ2-4VPS13B lines, they were observed in close proximity to the fibrillary inclusion bodies.

The accumulation of F-bodies in the cytoplasm of Δ2-4VPS13B cells near the ER and annulate lamellae can be explained by a cascade of events in which the loss of VPS13B triggers sequential disruptions of lipid transport, mitochondrial function, and proteostasis, ultimately leading to spatially organized accumulation of protein aggregates.

### 3.7. Transcriptome Profiling of VPS13B-Deficient Clones

To suggest possible mechanisms for the observed changes, we performed transcriptome analysis of Δ2-4VPS13B clones. Transcriptome analysis included eight samples (four controls; four samples with genetic modifications in *VPS13B*). A high correlation in gene co-expression was shown between all samples (>84%), and control samples displayed a correlation above 98%. Two modified samples—lines Δ2-4VPS13B#5 and Δ2-4VPS13B#24—were similar to control samples. However, there were two samples—Δ2-4VPS13B#4 and Δ2-4VPS13B#25—whose gene activity was less correlated with other samples, varying between 84% and 95% (Figure S2A). According to the principal component analysis (PCA) data, the first principal component explains 73% of the variance, and the most outlying sample on this component is Δ2-4VPS13B#25. Other samples are grouped along the first component but are dispersed along the second component (Figure S2B).

**Figure 10:**
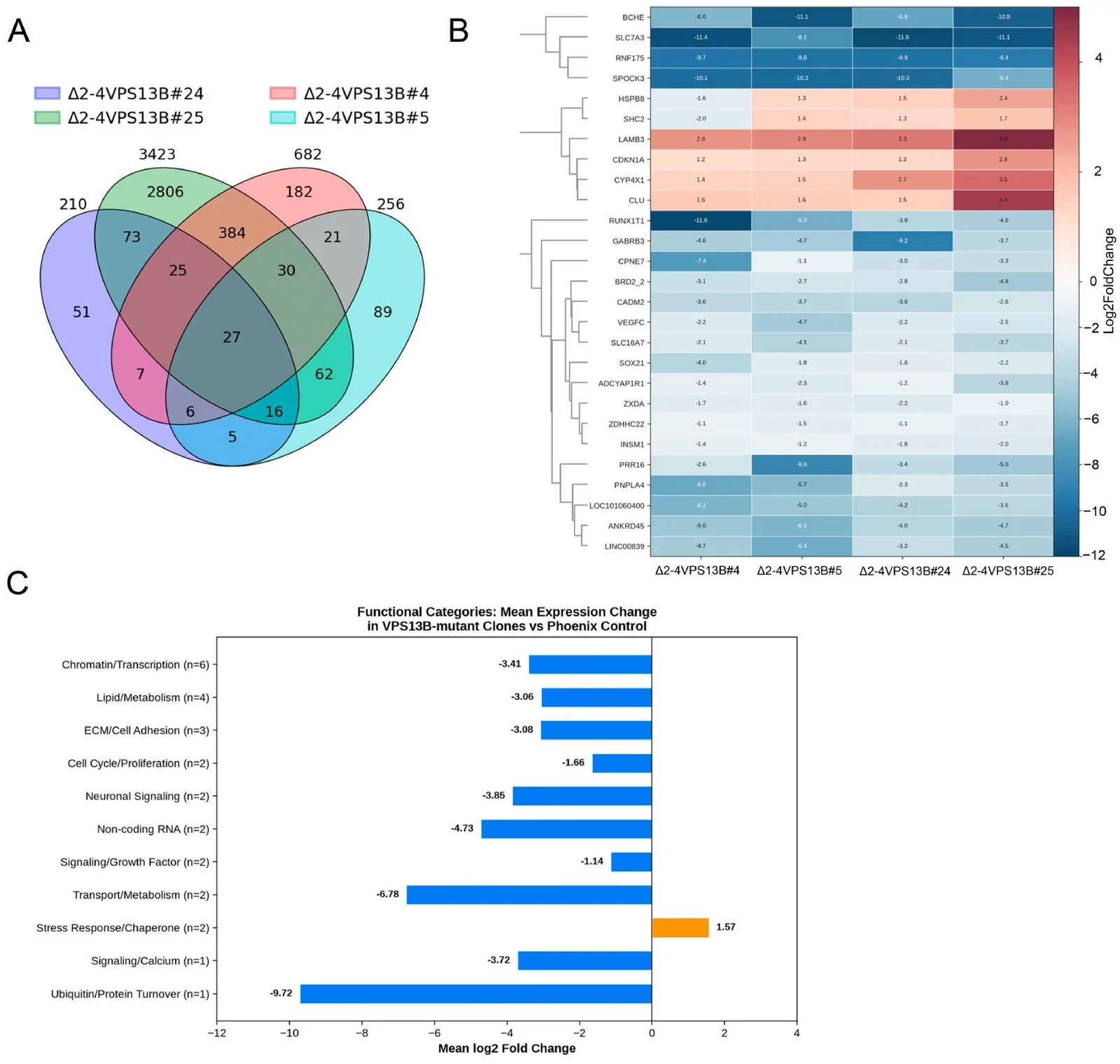
(**A**) Four-way Venn diagram showing the overlap of differentially expressed genes (DEGs) among the Δ2-4VPS13B#4, Δ2-4VPS13B#5, Δ2-4VPS13B#24, and Δ2-4VPS13B#25 lines. Numbers indicate the count of shared DEGs in each intersection. (**B**) Heatmap of Log2FoldChange values for 27 genes that are commonly expressed in all four samples (Δ2-4VPS13B#4, Δ2-4VPS13B#5, Δ2-4VPS13B#24, and Δ2-4VPS13B#25). Red color corresponds to upregulation, blue to downregulation. (**C**) Functional categories based on mean expression change in Δ2-4VPS13B clones in comparison with Phoenix control.

The analysis of differentially expressed genes (DEGs) in pairs of each sample and the control are presented in Supplemental Materials (Figure S3A–D). The DEG analysis in pairs of each sample and the control reveals that, in Δ2-4VPS13B#4 and Δ2-4VPS13B#5 clones, the majority of genes are downregulated (Log2FoldChange < −1), while, in Δ2-4VPS13B#24 and Δ2-4VPS13B#25, clones downregulated and upregulated genes are distributed equally. In addition, in the comparison of Δ2-4VPS13B#24 and Δ2-4VPS13B#25, it is noticeable that Δ2-4VPS13B#24 has a lower number of genes that change their activity. Overall, in line Δ2-4VPS13B#4, only 682 genes change their activity, 256 genes in line Δ2-4VPS13B#5, 210 genes in line Δ2-4VPS13B#24, and 3423 genes in line Δ2-4VPS13B#25 (Figure 10A). Comparison of the activity of specific genes revealed that the Δ2-4VPS13B clones show the lowest normalized read count in the Δ2-4VPS13B#25 clone. In the other samples, the normalized number of reads aligned to the *VPS13B* region is approximately equal to that observed in the control samples (Figure S1D).

We analyzed DEGs in four modified lines and examined the overlaps between genes showing |log2FoldChange| > 1. The largest overlap is concentrated at the intersection of Δ2-4VPS13B#25 and Δ2-4VPS13B#4 (384 genes). Δ2-4VPS13B#24 shares few common DEGs with the other samples: 7 with Δ2-4VPS13B#4, 5 with Δ2-4VPS13B#5, and 73 with Δ2-4VPS13B#25. All four samples possess a set of common DEGs comprising 27 genes (Figure 10A). Analysis of the activity of these 27 common genes (Figure 10B) reveals that the most robust decrease in activity across all four lines is observed for genes RNF175, SPOCK3, BCHE, and SLC7A3 (log2FoldChange < −6). Most genes show decreased activity, although CYP4X1, LAMP3, CDKN1A, and CLU exhibit log2FoldChange values above 1. Since sample Δ2-4VPS13B#25 is the most distinct, we analyzed Pearson’s correlation between Δ2-4VPS13B#25 and the other samples to identify uncorrelated genes. In all pairwise comparisons, we observed that RPS2, COX1, COX3, and EEF1A1 show activity that is uncorrelated between Δ2-4VPS13B#25 and the other samples (Figure S3E–G).

Functional analysis of differentially expressed genes (DEGs) using the ORA algorithm and GO term enrichment in the four samples revealed that Δ2-4VPS13B#4 shows the smallest variability in GO terms, with only four enriched categories. Most of these terms are related to extracellular structure organization. The Δ2-4VPS13B#24 line also has only four enriched groups, each containing more than 10 genes; these genes are involved in oxygen level detection and neuron projection extension. In contrast, Δ2-4VPS13B#5 and Δ2-4VPS13B#25 are enriched with a greater diversity of GO terms, although most of these genes are primarily responsible for synapse-related processes, including synapse organization, regulation of synapse organization, synapse assembly, and positive regulation of synapse assembly. When visualized as a GO-BP enrichment network, the significantly enriched terms organize into two interconnected functional clusters. The first cluster encompasses extracellular matrix and external encapsulating structure organization, alongside the regulation of neural precursor cell proliferation. The second, larger cluster comprises terms associated with nervous system development, synapse assembly and organization, muscle tissue development, locomotor behavior, response to xenobiotic stimuli, miRNA transcription regulation, and cellular response to retinoic acid. Notably, the network connectivity—in which GO terms sharing common genes are linked—reveals central hub terms that bridge synaptic regulation, miRNA metabolism, and retinoic acid signaling, coordinating multi-functional gene responses across structural and neurodevelopmental pathways. This pleiotropic architecture highlights key regulators belonging to multiple functional groups (Figure S4).

To identify functional gene groups among the 27 DEGs, we used the resource https://biomni.phylo.bio/, accessed on 1 July 2026 [32]. Notably, 21 of 27 genes (78%) were consistently downregulated across all four clones, while only 4 genes were consistently upregulated. The high inter-clone concordance (25 of 27 genes changed unidirectionally across all four clones) confirmed that these transcriptomic alterations are a reproducible consequence of *VPS13B* disruption rather than clone-specific artifacts. Functional annotation of the 27 DEGs using UniProt and NCBI Gene databases assigned them to 11 categories, of which 6 were most relevant to the established biology of VPS13B (Figure 10B,C).

The largest group comprised six genes involved in transcriptional regulation and chromatin remodeling (*BRD2*, *RUNX1T1*, *SOX21*, *INSM1*, *ANKRD45*, and *ZXDA*), all of which were downregulated. *BRD2* encodes a BET bromodomain protein that functions as an epigenetic reader of acetylated histones, linking chromatin to the transcriptional machinery [53]. *SOX21* and *INSM1* are transcription factors involved in neuronal differentiation [54]. The reduced expression of these genes suggests that VPS13B loss may compromise the delivery or function of transcriptional regulators, possibly secondary to disrupted vesicular trafficking and Golgi organization [12].

Four genes associated with lipid metabolism were altered: *BCHE* (log2FC = −8.7), *PNPLA4* (−4.5), and *ZDHHC22* (−1.4) were downregulated, while *CYP4X1* (+2.3) was upregulated. *ZDHHC22* encodes a DHHC palmitoyltransferase catalyzing protein S-palmitoylation, a modification critical for membrane targeting and vesicular transport. The alterations in this category are directly linked to the established role of VPS13B in delivering PI4P-enriched GA vesicles to mitochondrial fission sites and organelle contact sites [17].

Three genes related to extracellular matrix and cell adhesion were altered: *LAMB3* (log2FC = +3.5) was upregulated, while *CADM2* (−3.5) and *SPOCK3* (−9.2) were downregulated. *LAMB3* encodes the laminin beta-3 subunit, a component of basement membranes and hemidesmosomes. These changes are consistent with the role of VPS13B in tubular ERGIC formation and efficient procollagen export through the ERES–GA interface [12].

Four genes associated with stress response and cell cycle regulation showed induction of *CDKN1A* (p21/Cip1, log2FC = +1.6) and *CLU* (clusterin, +2.3), while *PRR16* (−5.0) was downregulated and *HSPB8* (+0.9) showed variable regulation. The concurrent induction of p21 and clusterin indicates activation of stress-induced premature senescence programs, consistent with the mitochondrial dysfunction described in VPS13B-deficient cells, including mitochondrial hyperfusion, decreased membrane potential, and impaired mitophagy [17].

Two genes involved in neuronal signaling were downregulated across all four clones: *GABRB3* (log2FC = −5.5), encoding the GABA-A receptor beta-3 subunit, and *ADCYAP1R1* (−2.2), encoding the PACAP receptor. Their suppression is consistent with the neuronal phenotypes of Cohen syndrome, including microcephaly and intellectual disability [1,55].

Two metabolite transporters were among the most strongly downregulated genes: *SLC7A3* (log2FC = −10.5), encoding CAT-3, a cationic amino acid transporter, and *SLC16A7* (−3.0), encoding MCT2, a monocarboxylate transporter. The pronounced suppression of these transporters may reflect altered metabolic requirements of cells with mitochondrial dysfunction secondary to VPS13B loss [17].

## 4. Discussion

To study the effect of disrupting VPS13B protein function, we used the CRISPR/Cas9 system with guide RNAs (gRNAs) targeting regions in the second and fourth exons to induce deletion of the segment of the *VPS13B* gene encoding the C-terminal domain of this protein in Phoenix cells (a derivative of the HEK293 cell line). As a result, we obtained five clones with a homozygous deletion of the genomic DNA between these gRNAs. Unexpectedly, in four of the five clones, alternative translation initiated from a start codon located in the fifth exon of the gene; consequently, these clones retained transcripts spanning the majority of the gene, with the exception of the deleted segment. However, in the fifth clone (Δ2-4VPS13B#25), nucleotide insertion occurred at the repair junction between the break sites, leading to a frameshift and nonsense-mediated decay (NMD). As a result, the read count mapping to *VPS13B* in this clone was reduced by 1.5-fold. When forming experimental groups, we generally did not account for this difference. In most cases, we investigated the changes induced by the *VPS13B* mutation that were common to all five clones, without emphasizing interclonal variations.

The first noticeable observation upon obtaining the mutant clones was a marked retardation in cell population growth. Indeed, calculation of the population doubling time revealed a significant increase of approximately 18% compared to the control Phoenix group. According to the literature, growth and developmental delay, as well as microcephaly, are characteristic features of patients with Cohen syndrome [1,55]. Furthermore, the animal model generated by Montillot et al. (2023) closely recapitulated the Cohen syndrome phenotype, demonstrating increased mortality, microcephaly, and growth retardation in newborn pups [56]. Reduced cell size has also been observed in neurons derived from patient-specific iPSCs with Cohen syndrome [18,19]. However, to the best of our knowledge, no studies have previously investigated growth parameters in the context of *VPS13B* mutation. Using the Δ2-4VPS13B clones, we demonstrated that the growth retardation occurs due to the G1 phase of the cell cycle, the duration of which is significantly prolonged in the mutant clones. A likely cause may involve the inactivation of genes participating in the regulation of transcription and chromatin remodeling, as well as increased transcription of the *CDKN1A* gene (p21/Cip1, log2FC = +1.6), which is a key mediator of cell cycle arrest and stress-induced senescence [57]. CDKN1A/p21 occupies a central position in the p53–p21–RB axis, where induction of p21 following DNA damage or other stress suppresses CDK activity, enhances the formation of RB–E2F repressor complexes, and leads to cell cycle arrest [58]. p21 not only triggers arrest but also often determines the transition to a persistent senescence-like state in response to genotoxic, telomeric, oncogenic, and metabolic stress [57,59,60].

Signs of cellular stress in the mutant clones were observed by electron microscopy, which revealed, in addition to the fragmented GA typically associated with *VPS13B* knockout, the presence of damaged mitochondria with membrane bulging and disruption, matrix-empty areas, and swollen cristae. This is consistent with recent findings on the involvement of VPS13B in mitochondrial division and quality control [17]. The authors demonstrated that the protein localizes to Mitofusin-2-positive mitochondria and recruits phosphatidylinositol-4-phosphate-rich GA-derived vesicles to sites of mitochondrial division, thereby supplying the lipids required for membrane scission. Loss of VPS13B leads to incomplete mitochondrial division and the accumulation of damaged, non-eliminated mitochondria within the cell. Beyond energy production, mitochondria are involved in the regulation of numerous cellular processes, including nuclear gene expression, Ca^2+^ homeostasis, cell stress, apoptosis, and necrosis; therefore, their normal structural organization, dynamics, and biogenesis are particularly critical for effective cellular function [61].

Balance maintenance between biogenesis and degradation of organelles is provided by components of the lytic system including lysosomes, autolysosomes, and autophagosomes and it is one of the important conditions for their functional activity and survival [62]. In mutant Δ2-4VPS13B#25 cells, the process of maturation of large autolysosomes was probably disrupted. The presence of clusters of lytic organelles of different sizes allows us to theorize that the *VPS13B* mutation altered the process of autolysosome formation and processing.

Furthermore, TEM analysis revealed ER abnormalities, manifested as dilation of the organellar lumen and alterations in its membranes, which appeared thickened, rigid, and inelastic. Additionally, we observed the appearance of fibrillary aggregates in the cytoplasm of mutant cells, which were not detected in control cells. Collectively, these findings may indicate proteolytic stress within the cell, resulting from impaired protein transport from the ER to the Golgi apparatus due to failed formation of tubular ERGIC—an atypical vesicular carrier that facilitates ER-to-Golgi transport [12,13]. This is partially confirmed by transcriptomic data, in which we detected an upregulation of *CLU* (Clusterin, +2.3 log2FC), a secreted glycoprotein with chaperone properties that protects cells from ER-stress-induced apoptosis [63,64]. It has been demonstrated that, under ER stress conditions, GRP78 (BiP) associates with CLU to facilitate its retrotranslocation and redistribution to the mitochondria, thereby reducing stress-induced apoptosis by cooperatively stabilizing mitochondrial membrane integrity [64]. CLU can form stable complexes with cytosolic misfolded proteins and direct them to the proteasome and autophagosomes for degradation [65]. Notably, CLU translocates to the cytoplasm, likely via a mechanism analogous to the ER-associated degradation (ERAD-like) pathway, and this process involves passage through the Golgi apparatus [66]. Interestingly, amyloid body accumulation may also arise from ERAD dysfunction [67]. Indeed, the amyloid-β precursor protein (APP), associated with Alzheimer’s disease, is a substrate of ERAD [68]. Studies indicate that, under ER stress, APP can be rapidly degraded via the ubiquitin–proteasome system, which is a component of ERAD.

However, an argument against the occurrence of ER stress via the unfolded protein response (UPR) upon *VPS13B* mutation is that the expression level of GRP78 (BiP), a classical chaperone that facilitates protein folding, prevents aggregation, and routes misfolded proteins toward degradation [69], remains unchanged both in our study and in earlier work [70]. Taking into consideration our findings of ER membrane morphology alterations, as well as the established role of VPS13B in regulating membrane lipid composition [38], we consider it plausible that ER stress in this context may be related to lipid bilayer stress (LBS) [71]. However, we realize that this interpretation remains speculative at this stage. Direct evidence, such as lipidomic analysis, membrane fluidity measurements, and assessment of LBS-specific signaling pathways, will be required to definitively establish this mechanism. Future studies are planned to address these questions.

Another finding is the altered localization of VPS13B at the GA, which we observed in Δ2-4VPS13B clones with a truncated C-terminal domain. It has previously been shown that VPS13B is a peripheral Golgi membrane protein that strongly co-localizes with the cis-Golgi matrix protein GM130 [36], although opinions regarding the mechanisms of its Golgi association remain highly controversial. Du et al. (2024) demonstrated the role of the PH domain at the C-terminus of the protein in targeting VPS13B to the Golgi through binding to phosphatidylinositol 4-phosphate on GA membranes [12]. However, Zorn et al. (2022) showed that GA localization of VPS13B was disrupted by disease-associated *VPS13B* missense variants located within, or rather in close proximity to, the VAB domain, whereas missense variants located closer to the Chorein/VPS13 region (aa 3–102, PFAM12624) and the beginning of a second VPS13 N-terminal region (aa 139–280), as well as those situated at the C-terminus of the protein, did not affect GA localization of VPS13B [10]. Notably, the position of the variants that direct VPS13B to the GA coincides with that of the Jellyroll fold module in VPS13B, which has been proposed to be responsible for the GA association of VPS13B [11]. The work by Du et al. further refines this mechanism; the PH domain alone is sufficient to target the Golgi, and its deletion from the full-length protein abolishes this capacity, directly linking disruption of the domain structure to the loss of Golgi localization [12]. The same study demonstrates that VAB missense mutations disrupt the interaction between VPS13B and Sec23IP, indicating that structural defects may impair recruitment of the protein to the ERES–Golgi interface. However, none of these studies reported a change in VPS13B localization upon mutation of the DNA region encoding the N-terminal domain.

Also noteworthy is the change in protein localization under NMD, in which antibodies against the protein concentrate immediately beneath the plasma membrane. Seifert et al. (2014) reported that COH1 localization at the GA depends on RAB6 and that, upon RAB6A/A′ knockdown, COH1 fails to localize to the Golgi; however, this loss of localization results from impaired recruitment rather than from an alteration in the intrinsic structure of VPS13B [15].

## 5. Conclusions

Using CRISPR/Cas9-mediated deletion of the VPS13B region encoding the C-terminal domain in Phoenix cells, we generated five homozygous mutant clones and provide the first direct cellular evidence that VPS13B loss recapitulates the growth retardation characteristic of Cohen syndrome. Mutant clones showed an increase in population doubling time driven by prolonged G1 phase, consistent with upregulation of CDKN1A/p21 and activation of the senescence-like program. Beyond proliferation, VPS13B disruption produced broad organelles dysfunction—fragmentation of the GA, structurally damaged mitochondria with impaired division, defective autolysosome maturation, and ER membrane abnormalities accompanied by cytoplasmic fibrillary aggregates. These all indicate a proteolytic and lipid-bilayer stress arising from failed ER-to-Golgi transport rather than a canonical unfolded protein response. We further show that C-terminal truncation abolishes VPS13B Golgi localization, whereas nonsense-mediated decay relocalizes residual protein to the cytoplasmic membrane region, expanding our understanding of protein localization of VPS13B. Together, these data indicate that loss or structural alteration of VPS13B perturbs membrane lipid handling, organelle dynamics, and proteostatic pathways, which result in cell-cycle arrest and cellular stress, the phenotypes that highlight the role of VPS13B in maintaining Golgi–ER–mitochondrial homeostasis.

## Data Availability

The original data presented in the study are openly available in NCBI’s Sequence Read Archive (SRA) http://www.ncbi.nlm.nih.gov/bioproject/1492069, accessed on 8 July 202 at accession number PRJNA1492069.

## Supplementary Materials

The following supporting information can be downloaded at: https://www.mdpi.com/article/10.3390/cells15171535/s1.
