# Supplementary material for: Modeling Cohen Syndrome in Phoenix Cells: VPS13B Loss Causes Organelle Stress, G1/S Delay, and Fibrillary Inclusion Bodies Formation
Source: Cells. 2026 Aug 26;15(17):1535. doi: 10.3390/cells15171535 (PMC13564750; doi:10.3390/cells15171535)
Supplement: Supplementary file 1 [file cells-15-01535-s001.zip › cells-4460028-supplementary.pdf]

Supplementary Information

Table S1. List of used primers and gRNA

Primers	Target	Forward/Reverse primer (5′-3′)	product
exon 2 gRNA		5'-CTCCTTACCTTAAAAGATGC-3'	
exon 4 gRNA		5'-GGTGGTAAGTCAGGATCTGT-3'	
Targeted mutation analysis (PrCOH1 2-4ex)	2-4 exons	CTCTACCGCCGACTTCTAACGT/ CACTTCCAGCCTGGGGATATT	773bp
Targeted mutation analysis (PrCOH1 3-4ex)	3-4 exons	TATCTCCTGTAGCTACCATAAACTG/ CACTTCCAGCCTGGGGATATT	836bp
RT-PCR PrCOH1 2-4ex		GTGGAGACGTGGTACTCAGC/ GCATTCTCCGCGGTTTGATT	282bp
RT-PCR PrCOH1 25-28ex F	25-28 exons	AGGCACAGCTCCTCCAGATA/ ATGTCCTGACTGCCAACCTT	379bp
House-keeping gene (RT-PCR)	GPDH	GTGGACCTGACCTGCCGTCT/ GGAGGAGTGGGTGTCGCTGT	153 bp

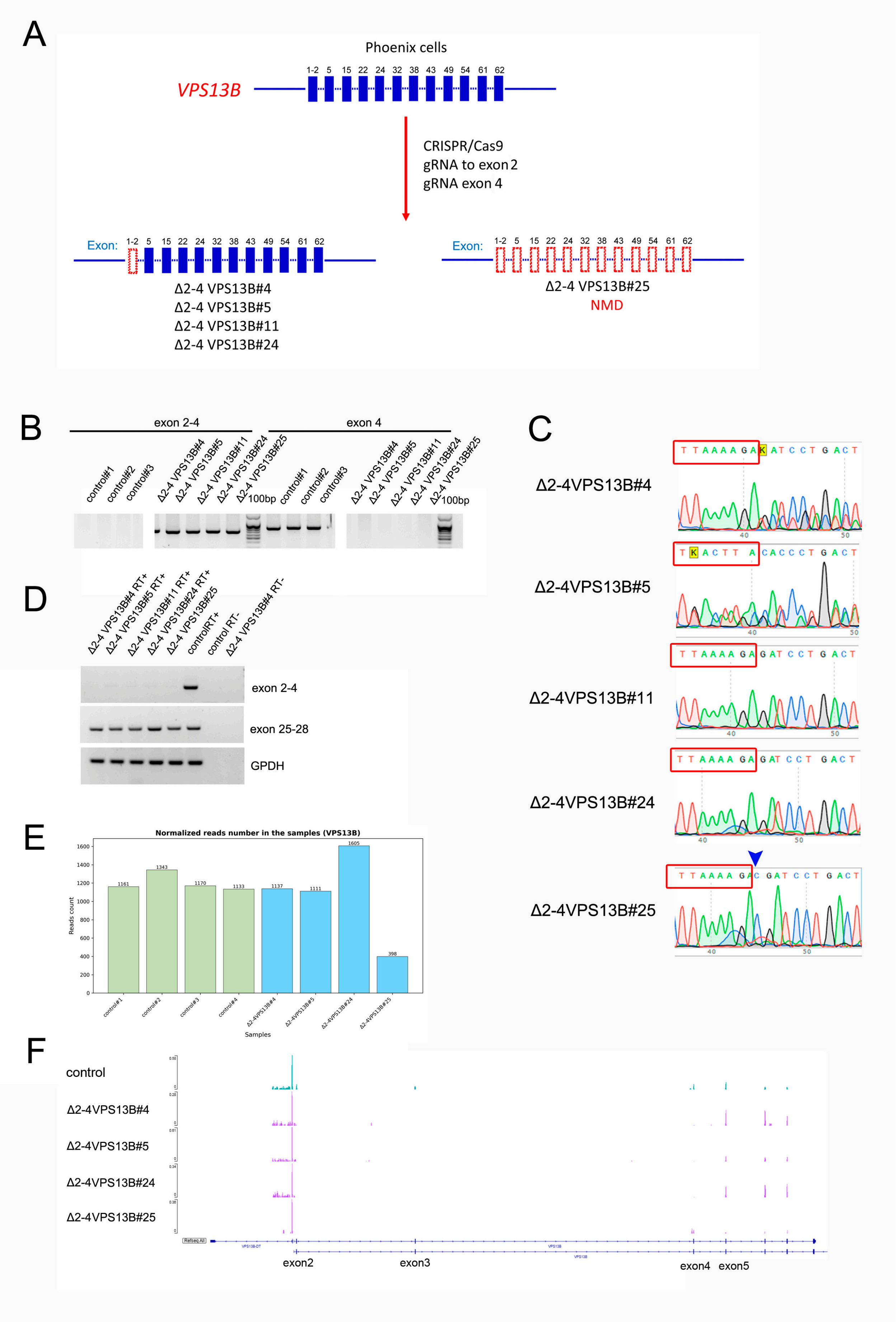

Figure S1. Generation and characteristics of mutant Phoenix cell line.

(A) The five independent clones carrying *VPS13B* mutations were generated using CRISPR/Cas9 system with guide RNAs. Four clones harboring a disrupted DNA segment within exons 2 - 4 and one clone exhibiting probable nonsense-mediated mRNA decay (NMD).

(B) PCR amplification of the product of exons 2–4 deletion using flanking primers (2-4 exon) and the exon 4 region.

(C) Sanger sequencing of *VPS13B* in the Δ2-4VPS13B clones. Arrows indicate the insertion of one nucleotide in Δ2-4VPS13B#25 clone. The red box marks the DNA region upstream of the break in exon 2

(D) RT-PCR product of *VPS13B* in the region of exon 2-4 and exon 25-28.

(E) Normalized reads number in the Phoenix controls and Δ2-4 VPS13B samples clones. Read counts for the VPS13B gene, normalized to transcripts per million (TPM).

(F) RNA-seq coverage tracks (CPM-normalized, 10 bp bins) across the *VPS13B* locus (chr8:99,009–99,116 kb, GRCh38) in Phoenix control and four CRISPR-edited clones.

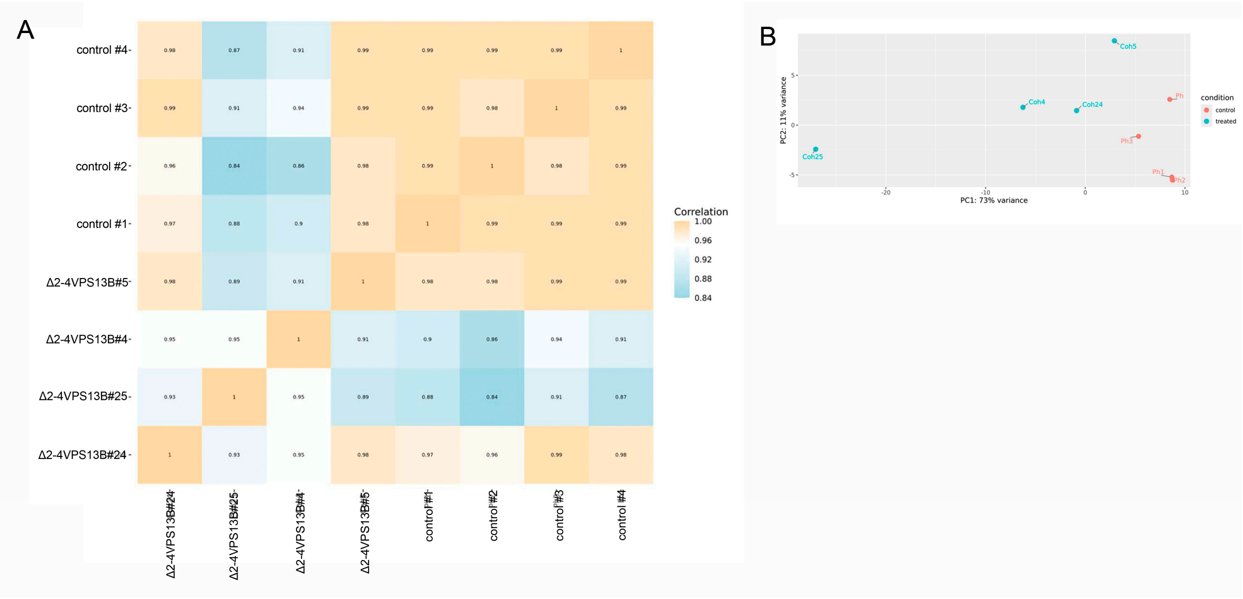

Figure S2. Correlation of gene activity and PCA analysis.

(A) Correlation of genes activity in 8 samples included in the transcriptome analysis. (B) PCA analysis of the samples, red points correspond to control samples and blue points correspond to the Δ2-4VPS13B samples.

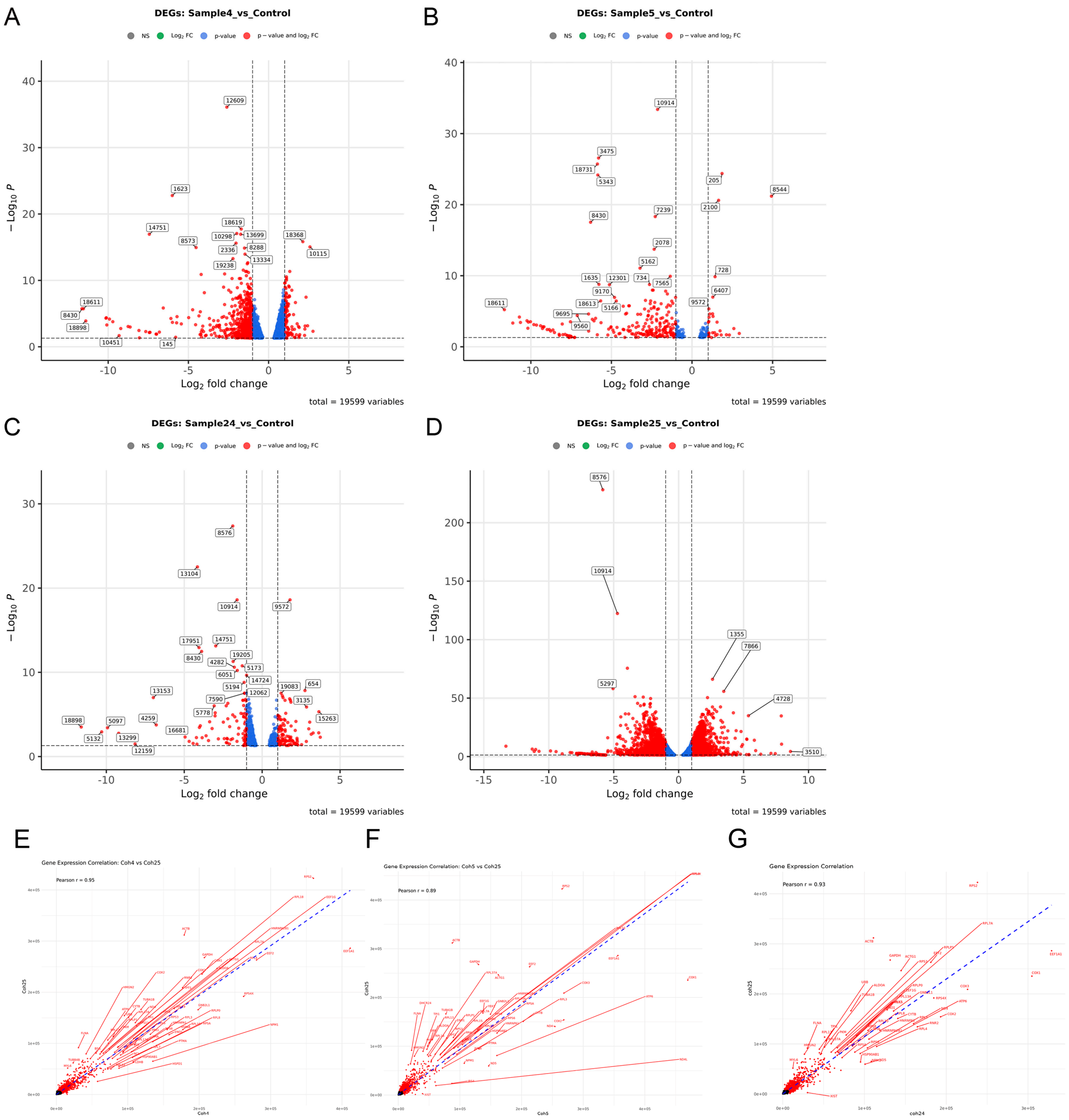

Figure S3. Gene expression analysis between the control sample and each of the four modified lines.

Volcano plots showing differential gene expression analysis between the control sample and each of the four modified lines: Δ2-4VPS13B#4, Δ2-4VPS13B#5, Δ2-4VPS13B#24, and Δ2-4VPS13B#25 (A–D, respectively). Each point represents a gene. The X-axis shows the $\log_2$FoldChange, with dashed vertical lines at –1 and 1. The Y-axis shows the $-\log_{10}$(p-value), with a dashed horizontal line at 1.30, corresponding to a nominal p-value of 0.05. Pearson correlation of gene expression between Δ2-4VPS13B#25 and Δ2-4VPS13B#4 (r=0.95) (E). Δ2-4VPS13B#25 and Δ2-4VPS13B#5 (r=0.89) (F), between Δ2-4VPS13B#25 and Δ2-4VPS13B#24 (r=0.93) (H).

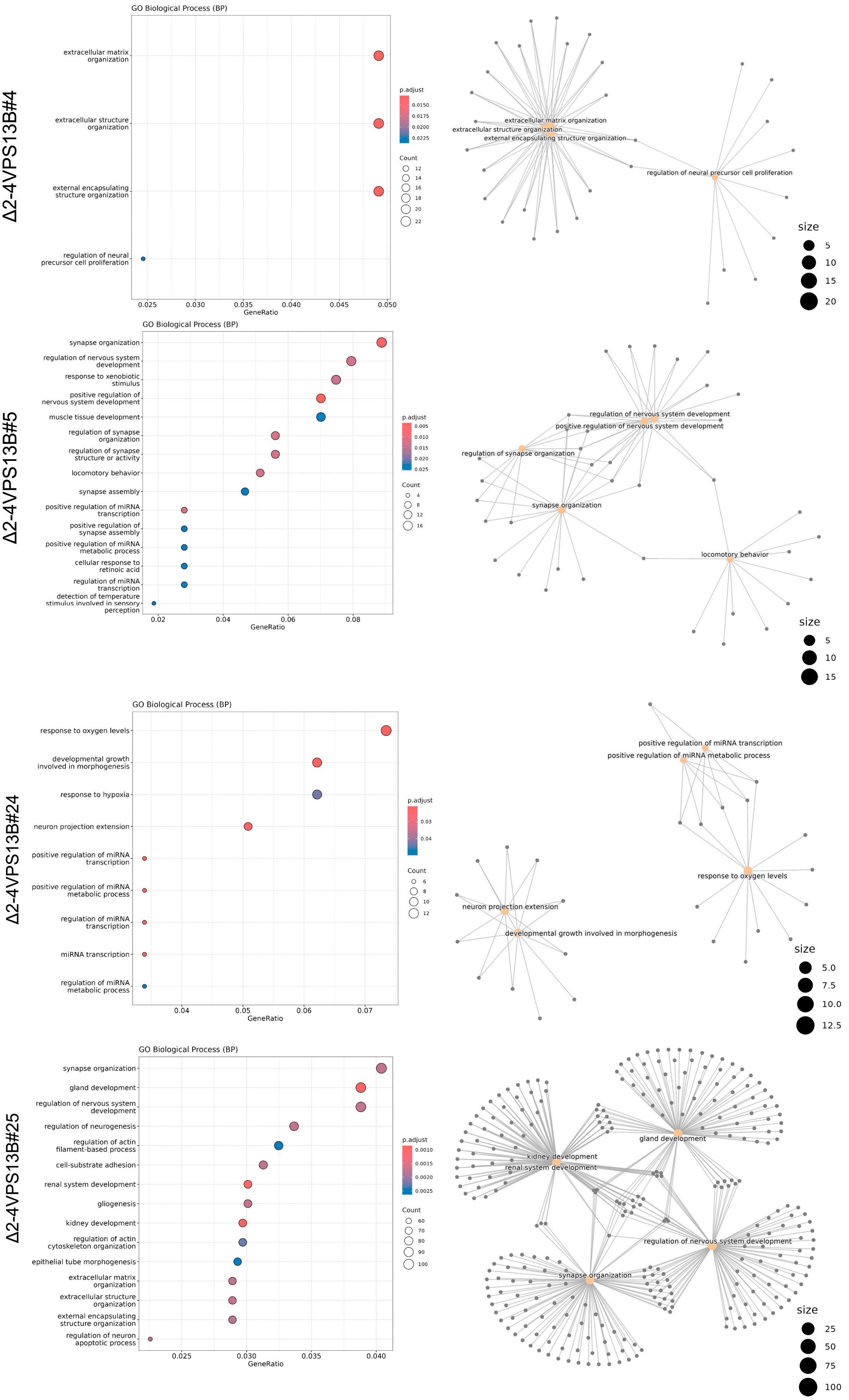
Δ2-4VPS13B#4
GO Biological Process (BP)
extracellular matrix organization
extracellular structure organization
external encapsulating structure organization
regulation of neural precursor cell proliferation
GeneRatio
p.adjust
Count
regulation of neural precursor cell proliferation
size
Δ2-4VPS13B#5
GO Biological Process (BP)
synapse organization
regulation of nervous system development
response to xenobiotic stimulus
positive regulation of nervous system development
muscle tissue development
regulation of synapse organization
regulation of synapse structure or activity
locomotory behavior
synapse assembly
positive regulation of miRNA transcription
positive regulation of synapse assembly
positive regulation of miRNA metabolic process
cellular response to retinoic acid
regulation of miRNA transcription
detection of temperature stimulus involved in sensory perception
GeneRatio
p.adjust
Count
size
Δ2-4VPS13B#24
GO Biological Process (BP)
response to oxygen levels
developmental growth involved in morphogenesis
response to hypoxia
neuron projection extension
positive regulation of miRNA transcription
positive regulation of miRNA metabolic process
regulation of miRNA transcription
miRNA transcription
regulation of miRNA metabolic process
GeneRatio
p.adjust
Count
size
Δ2-4VPS13B#25
GO Biological Process (BP)
synapse organization
gland development
regulation of nervous system development
regulation of neurogenesis
regulation of actin filament-based process
cell-substrate adhesion
renal system development
gliogenesis
kidney development
regulation of actin cytoskeleton organization
epithelial tube morphogenesis
extracellular matrix organization
extracellular structure organization
external encapsulating structure organization
regulation of neuron apoptotic process
GeneRatio
p.adjust
Count
size

Figure S4. Enrichment network of Gene Ontology Biological Processes (GO-BP) among differentially expressed genes.

Over-representation analysis (ORA) results visualized as an enrichment network of significantly enriched GO-BP terms. Node color gradients correspond to adjusted p-value with darker hues indicating higher significance, node sizes reflect gene count per term. Edges connect GO terms sharing common genes.
